# Decoding the Reference Letter: Strategies to Reduce Unintentional Gender Bias in Letters of Recommendation

**DOI:** 10.15766/mep_2374-8265.11419

**Published:** 2024-07-05

**Authors:** Bethel R. Mieso, Jonathan F. Barnett, Tiffany M. N. Otero, Sean W. Berquist, Felipe D. Perez, Peggy Han, Sumit Bhargava, Anaid Atasuntseva, Lahia Yemane

**Affiliations:** 1 Third-Year Pediatrics Resident, Department of Pediatrics, Stanford University School of Medicine; 2 Clinical Assistant Professor, Division of Pediatric Anesthesiology, Department of Anesthesiology, Perioperative and Pain Medicine, Stanford University School of Medicine; 3 Anesthesiologist and Intensivist, Department of Anesthesiology, Ochsner Medical Center; 4 Sixth-Year Urology Resident, Department of Urology, Stanford University School of Medicine; 5 Clinical Associate Professor, Division of Pediatric Anesthesiology, Department of Anesthesiology, Perioperative and Pain Medicine, Stanford University School of Medicine; 6 Clinical Associate Professor, Division of Critical Care Medicine, Department of Pediatrics, Stanford University School of Medicine; 7 Clinical Professor, Division of Pediatric Pulmonology & Sleep Medicine, Department of Pediatrics, Stanford University School of Medicine; 8 Clinical Instructor, Division of Child and Adolescent Psychiatry, Department of Psychiatry and Behavioral Sciences, Stanford University School of Medicine; 9 Clinical Associate Professor, Division of General Pediatrics, Department of Pediatrics, Stanford University School of Medicine

**Keywords:** Gender Bias, Admissions/Selection, Bias, Faculty Development, Gender Issues in Medicine, Promotions & Tenure, Diversity, Equity, Inclusion

## Abstract

**Introduction:**

There is a growing body of literature on gender bias in letters of recommendation (LORs) in academic medicine and the negative effect of bias on promotion and career advancement. Thus, increasing knowledge about gender bias and developing skills to mitigate it is important for advancing gender equity in medicine. This workshop aims to provide participants with knowledge about linguistic bias (focused on gender), how to recognize it, and strategies to apply to mitigate it when writing LORs.

**Methods:**

We developed an interactive 60-minute workshop for faculty and graduate medical education program directors consisting of didactics, reflection exercises, and group activities. We used a postworkshop survey to evaluate the effectiveness of the workshop. Descriptive statistics were used to analyze Likert-scale questions and a thematic content analysis for open-ended prompts.

**Results:**

We presented the workshop four times (two local and two national conferences) with one in-person and one virtual format for each. There were 50 participants who completed a postworkshop survey out of 74 total participants (68% response rate). Ninety-nine percent of participants felt the workshop met its educational objectives, and 100% felt it was a valuable use of their time. Major themes described for intended behavior change included utilization of the gender bias calculator, mindful use and balance of agentic versus communal traits, closer attention to letter length, and dissemination of this knowledge to colleagues.

**Discussion:**

This workshop was an effective method for helping participants recognize gender bias when writing LORs and learn strategies to mitigate it.

## Educational Objectives

By the end of this workshop, participants will be able to:

1. Define linguistic bias and gender bias in letters of recommendation (LORs).
2. Identify adjectives in LORs commonly associated with gender bias.
3. Apply tools learned to mitigate gender bias when writing LORs.
4. Discuss strategies to reduce gender bias when writing LORs.

## Introduction

Letters of recommendation (LORs) are a key part of career advancement in academic medicine, yet there is significant variability in how they are constructed. Residency and fellowship program directors agree that LORs are one of the most important factors in selecting applicants for interviews,^1,2^ and an individual's candidacy for a program may be significantly impacted by the language used in their LOR.^3^ Unfortunately, a growing body of literature highlights gender bias in LORs that can negatively impact promotion and advancement.

Given the historic social roles defined for both men and women, society tends to view men as agentic individuals described as assertive, independent, and confident, while women are perceived to be communal individuals described as helpful, caring, and interpersonal.^4–6^ Agentic and communal language have their roots in social role theory, reinforcing gender stereotypes,^4^ and these biases are seen in LORs with male applicants more often described using agentic terms while female applicants are more often described using communal terms.^5–7^ LORs for male applicants are also often longer in length, more likely to reference their research and accomplishments, and less likely to have minimal assurance language such as “he/she can do the job” or doubt-raiser language such as “while not the best person I've worked with.”^8,9^ In addition, standout adjectives such as *excellent, outstanding,* and *exceptional* are used with greater frequency in letters written for male applicants than for female applicants.^9,10^

Many studies have shown the impact of gender bias in LORs on career advancement across multiple medical specialties, including female-predominant specialties.^5,6,11–15^ Studies indicate that applicants with invitations to interview at residency programs had LORs that were longer in length and contained more standout adjectives and research words as compared to those without interview invitations.^16^ Similarly, among urology resident applicants, words associated with power such as *superior* as defined by the Linguistic Inquiry and Word Count analytic program were used significantly more for male applicants than female applicants and were also more associated with LORs for applicants who had matched into urology.^13^ Importantly, gender-based language differences have not been shown to correlate to USMLE scores.^11,13^ Thus, gender-based linguistic differences in LORs can significantly influence career advancement for female applicants.^5,14,17^

Most letter writers have not received guidance or formal training on writing LORs, highlighting an important educational gap.^18^ With more literature showing gender, racial, and ethnicity biases in LORs, there have been calls across multiple specialties for standardization of LORs.^19,20^ *MedEdPORTAL* has several examples of workshops that aim to help trainees and faculty recognize and address implicit biases broadly and/or towards specific groups or identities, but currently there are no *MedEdPORTAL* publications that specifically address the topic of gender bias in LORs and strategies to mitigate it. This interactive workshop aims to provide participants with knowledge about linguistic bias (focused on gender), how to recognize it, and how to apply strategies to mitigate it when writing LORs.

## Methods

### Facilitators

We initially developed this workshop as a diverse group of individuals—medical residents, fellows, and faculty from various specialties—who participated in the Stanford Medicine Leadership Education in Advancing Diversity (LEAD) Program from 2021 to 2022. We had two to five facilitators available to present each workshop, and this variability was dependent on audience size and facilitator availability. Each facilitator was familiar with the entire workshop and therefore able to lead any part of it as needed. No specialized training was required to facilitate the workshop aside from review of the materials to be familiar with the content.

### Target Audience

The target audience for the workshop was faculty reading and/or writing LORs in academic medicine, including clinical and research faculty as well as residency and fellowship program directors.

### Workshop

We used Kern's six-step model to systematically design, implement, and evaluate the workshop.^21^ The first and second steps of problem identification and targeted needs assessment led us to the topic of gender bias in LORs through careful literature review and discussion with the author team and LEAD coparticipants. The third step—outlining goals and objectives—was performed by identifying the gaps in the literature. The fourth step included aligning the goals and objectives with an interactive workshop format that featured brief didactics, reflection exercises, and small- and large-group activities. The fifth step involved presenting the workshop at several conferences. Lastly, the sixth step used a postworkshop survey to evaluate the effectiveness of the workshop in meeting its educational objectives and participants’ plans for intended behavior change.

We initially developed a 75-minute, in-person workshop; however, we adapted the workshop to fit various times allotted for sessions, different formats (in person vs. virtual), and feedback from postworkshop evaluations. Major adaptations included shortening the introductory reflection exercises (e.g., we removed a riddle and video that highlighted gender bias) and shortening the length of the didactic portions to allow for more interactive components. Adaptations made for the 60-minute virtual sessions included replacing small- and large-group activities with the chat and unmute feature to avoid excessive utilization of time needed for multiple breakout room transitions.

We presented the workshop via PowerPoint format (Appendix A) using the agenda outlined in the facilitator guide (Appendix B). We began with introductions, sharing the educational objectives, and acknowledging that the literature and discussion of gender bias in LORs in academic medicine might not apply to the full spectrum of gender as it primarily focused on binary gender (man vs. woman). We then led a reflection activity in which participants were asked to compare two excerpts from LORs, with names and pronouns redacted (Appendix C), which we would return to later in the workshop. Participants were asked to answer reflection questions about the similarities and differences noticed. This activity was followed by a brief didactic outlining the relevance of the workshop topic. Next, we led a short activity in which participants listed personal characteristics that they valued in themselves. Afterwards, there was a brief didactic on agentic versus communal traits, and participants were asked to refer back to their personal characteristics and categorize them. During the in-person workshops, participants wrote these on post-it notes and placed them on large sheets labeled *agentic* and *communal* as a visual representation of the groups’ responses. This was followed by a thoughtful large-group reflection and discussion on what traits were valued more in medicine and why.

We began the discussion of individual strategies to mitigate gender bias by sharing examples of gender-biased language and introducing the gender bias calculator tool.^22^ We received written permission to use the calculator in this presentation and future dissemination from its creator, Thomas Forth. Although there were multiple gender bias tools available, our group decided to use this specific gender bias calculator because it was created specifically for gender bias found in LORs and was offered free to the public. We then reintroduced the LOR excerpts from the beginning of the workshop, now with names and pronouns revealed (Appendix D). Next we presented the results of the gender bias calculator, using the example letters to highlight the use of agentic versus communal terms (Appendix E), and reflection questions were discussed. We concluded the workshop by describing strategies to mitigate gender bias in writing LORs, including a one-page tip sheet we had created (Appendix F). We reserved the final 5 minutes for questions and for participants to complete the postworkshop evaluation (Appendix G). We provided supplementary handouts for Appendices C–E, which complemented the PowerPoint slides, to enhance overall readability.

### Evaluation and Analysis

At the conclusion of the workshop, we asked participants to complete an anonymous postworkshop evaluation (Appendix G), which was an electronic link or in paper form depending on the presentation format. The survey was used to evaluate the effectiveness of the workshop in meeting the educational objectives using a 5-point Likert scale (1 = *strongly disagree,* 5 = *strongly agree*) and open-ended questions to explore anticipated behavior change, what had worked well, and what could be improved in the future. To analyze the results, we used descriptive statistics, and two authors (Bethel R. Mieso and Lahia Yemane) coded open-ended responses using conventional content analysis.

### Institutional Review Board

This study was submitted for review to the Stanford University Institutional Review Board and was determined not to meet the definition of human subject research (protocol number: 67884).

## Results

We presented the workshop four times (twice virtually, twice in person), including two local conferences (Stanford Medicine 5th Annual Diversity & Inclusion Forum, June 2022; Stanford Department of Pediatrics Professional Development Series, June 2023) and two national conferences (Building the Next Generation of Academic Physicians, March 2023; Association of Pediatric Program Directors Annual Conference, March 2023). Participants at the sessions were primarily faculty given the relevance of this topic and the fact that two of the conferences (Association of Pediatric Program Directors and Department of Pediatrics Development Series) were geared toward faculty and educational leaders. There were 50 participants who completed a postworkshop evaluation form out of 74 total participants (68% response rate).

Of the participants who completed the postworkshop evaluations, 99% somewhat or strongly agreed that the workshop met all of its educational objectives, and 100% felt it was a valuable use of their time (Table 1). Major themes participants described for intended behavior change included application of the gender bias calculator, mindful use and balance of agentic versus communal traits, closer attention to letter length, and dissemination of this new knowledge to colleagues. Respondents also noted that the main barriers to applying the lessons learned in the workshop were time constraints and institutional buy-in (Table 2). Aspects the participants liked best about the workshop were its interactive nature, using sample LORs in activities, the reflection exercises, and the tip sheet provided.

**Table 1.**
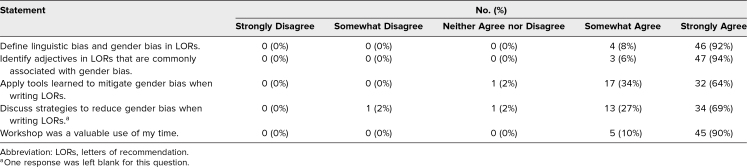
Participant Responses (*N* = 50) to Postworkshop Evaluation

**Table 2.**
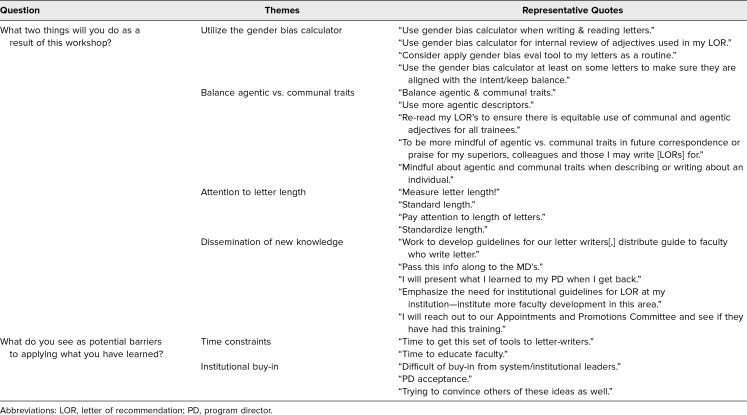
Participant Responses Regarding Intended Behavior Change and Barriers to Implementation

## Discussion

We designed and implemented an interactive workshop to provide participants with knowledge about linguistic bias (focused on gender) and how to recognize it, as well as strategies to mitigate it when writing LORs. Evaluations from participants showed that the workshop was effective in meeting its educational objectives, provided tangible strategies for behavior change, and addressed an important educational gap.

During the four workshops, participants had rich discussions about the tension of agentic versus communal terms used to describe applicants and how those may be valued differently by various individuals, specialties, and so on. While we recognize that both agentic and communal traits are valuable in academic medicine, the literature reveals that the culture of medicine tends to value agentic traits to a greater extent.^4,17^ In a study by Brown and colleagues, women letter writers for obstetrics and gynecology residency applicants used communal terms in LORs more often than men letter writers; interestingly, women letter writers used communal terms at the same frequency in letters written for both male and female applicants.^17^ This may demonstrate that women letter writers value communal traits differently than men letter writers. Given this, it is important to strike a balance in the use of these adjectives when describing an applicant of any gender with the understanding that both are important and necessary traits for physicians. Questions were raised about how to recognize bias from the reader's perspective given that many letter writers often also read LORs. Although the focus of this workshop is on writing LORs, we feel that the same knowledge is applicable as a reader.

In the workshop, we highlight the importance of intentional design and construction of LORs, in which language plays a significant role. LORs written for female applicants tend to be shorter; have fewer praise words, fewer power words, less agentic language, and less mention of research or skills; and include more doubt raisers as compared with LORs for male applicants.^5–10,13^ LORs for applicants invited to interview or for matched applicants are longer and have more standout, power, ability, and research words than LORs for applicants not invited to interview, features that tend to be included less frequently in LORs written for women.^13,16^ This demonstrates the negative impacts that gender bias in LORs can have on career advancement for female applicants and negative consequences on advancing equity in medicine.^5,14,17^

As the literature suggests, faculty development activities to increase awareness of gender-biased language, their interpretations, and negative impacts are a necessary first step.^3,14,23^ These can take place in the form of workshops, like the one described here, as well as antibias training sessions. Second, research has described guidelines and standardization of letters as ways to mitigate gender bias at the system level.^12,16,19,23^ Zhang, Blissett, Anderson, O'Sullivan, and Qasim demonstrated that the Alliance for Academic Internal Medicine's guidelines to standardize program director letters for fellowship helped mitigate gender bias.^23^ Similarly, a study by Friedman and colleagues showed that the standardized LOR adopted by otolaryngology head and neck surgery residency programs reduced gender bias in LORs.^12^ Emphasis on competency-based performance has also been described as a strategy to reduce gender bias in LORs.^14,17,23^

We presented this workshop in virtual and in-person formats and different time allotments, and all were found to be effective. For the virtual workshop, we replaced the breakout groups in the in-person format with the Zoom chat feature and large-group discussion. We developed iterations of the workshop based on feedback from the previous presentation. Having both virtual and in-person formats available offers flexibility based on the accessibility needs of participants. From our perspective as presenters, the virtual format tends to be more engaging when the presentation is given to a group of individuals more familiar with each other. In-person formats allow for more interactive and visual activities and provide the opportunity for longer reflection with small breakout groups.

Limitations include the focus on binary gender bias and not addressing intersectionality, which we mention at the beginning of the workshop in framing the session. We acknowledge that there is a lack of literature on nonbinary gender bias in LORs, an important area for future work. The evaluation response rate of 68% was lower than anticipated and may not reflect the perspectives of participants who did not complete the postworkshop evaluation. We did not collect participant demographic information (i.e., gender, educational role, career stage), which could have influenced participants’ perception of the workshop's relevance. Our evaluation assessed participants’ perception of whether the workshop met its stated educational objectives; however, we did not include specific questions to assess knowledge and skills gained. Future iterations could consider allowing additional time or follow-up sessions for further skill application. Although participants shared intended behavior change on the evaluation, a long term follow-up evaluation at 3–6 months would be important to understand the true impact of this workshop and assess its influence on actual future practice.

LORs play an important role in the application process for residency, fellowship, and faculty positions. Therefore, recognizing gender-based linguistic differences and applying strategies to mitigate such bias in writing LORs are critical for advancing equity in academic medicine.


Decoding the Reference Letter Presentation.pptxFacilitator Guide.docxExample Letters - Redacted Version.docxExample Letters - Unredacted Version.docxGender Bias Calculator With Example Letters.docxStanford LOR Tip Sheet.pdfWorkshop Evaluation Form.doc
*All appendices are peer reviewed as integral parts of the Original Publication.*
