# Supplementary material for: Decoding the Reference Letter: Strategies to Reduce Unintentional Gender Bias in Letters of Recommendation
Source: MedEdPORTAL. 2024 Jul 5;20:11419. doi: 10.15766/mep_2374-8265.11419 (PMC11224141; doi:10.15766/mep_2374-8265.11419)
Supplement: Supplementary file 1 — Decoding the Reference Letter Presentation.pptxFacilitator Guide.docxExample Letters - Redacted Version.docxExample Letters - Unredacted Version.docxGender Bias Calculator With Example Letters.docxStanford LOR Tip Sheet.pdfWorkshop Evaluation Form.doc [file mep_2374-8265.11419-s001.zip › A. Decoding the Reference Letter Presentation.pptx]

## Slide 1
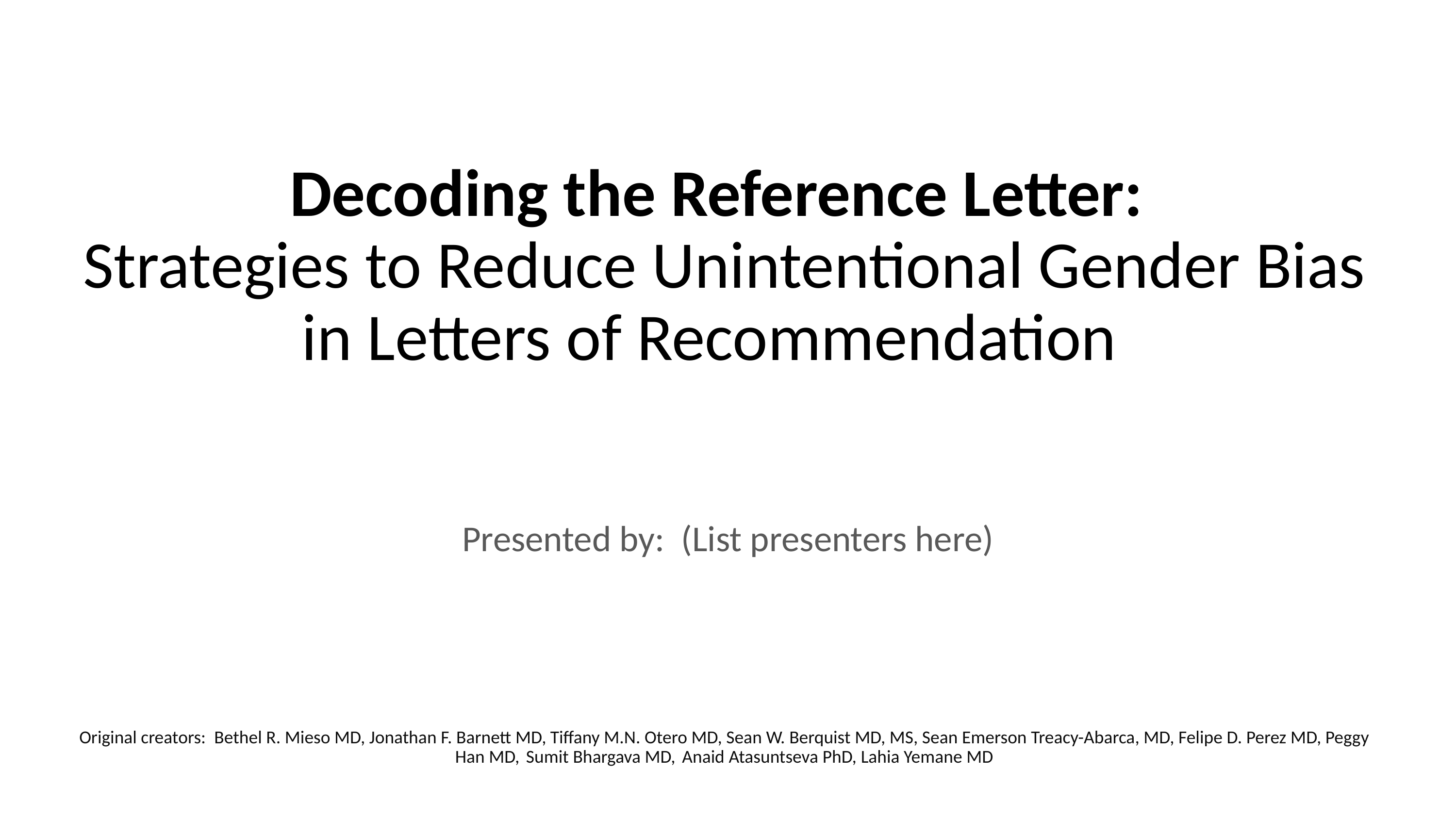

# Decoding the Reference Letter:  Strategies to Reduce Unintentional Gender Bias in Letters of Recommendation
Presented by: (List presenters here)
Original creators:  Bethel R. Mieso MD, Jonathan F. Barnett MD, Tiffany M.N. Otero MD, Sean W. Berquist MD, MS, Sean Emerson Treacy-Abarca, MD, Felipe D. Perez MD, Peggy Han MD,  Sumit Bhargava MD,  Anaid Atasuntseva PhD, Lahia Yemane MD

## Slide 2
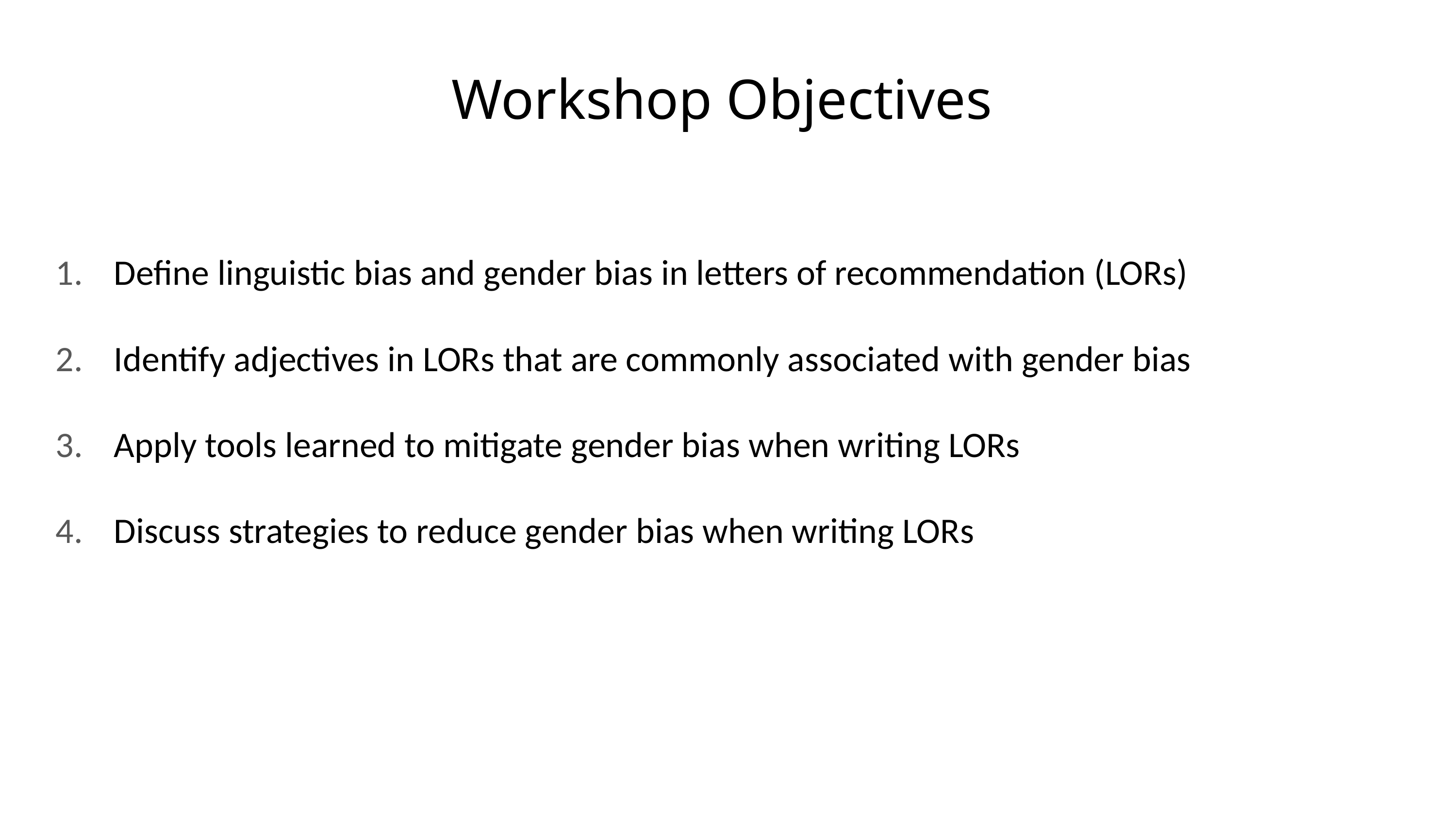

# Workshop Objectives
Define linguistic bias and gender bias in letters of recommendation (LORs)
Identify adjectives in LORs that are commonly associated with gender bias
Apply tools learned to mitigate gender bias when writing LORs​
Discuss strategies to reduce gender bias when writing LORs

## Slide 3
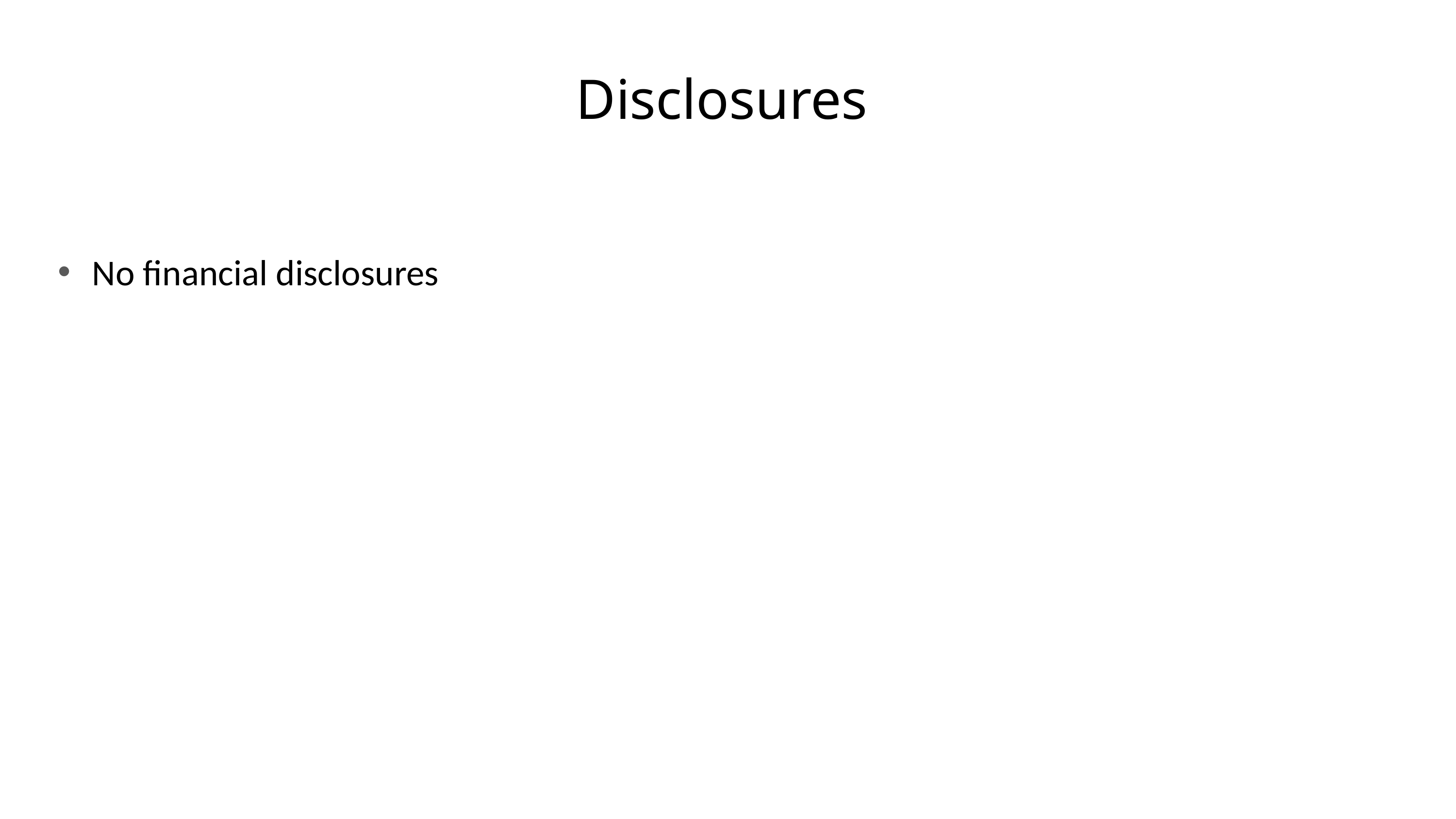

# Disclosures
No financial disclosures​

## Slide 4
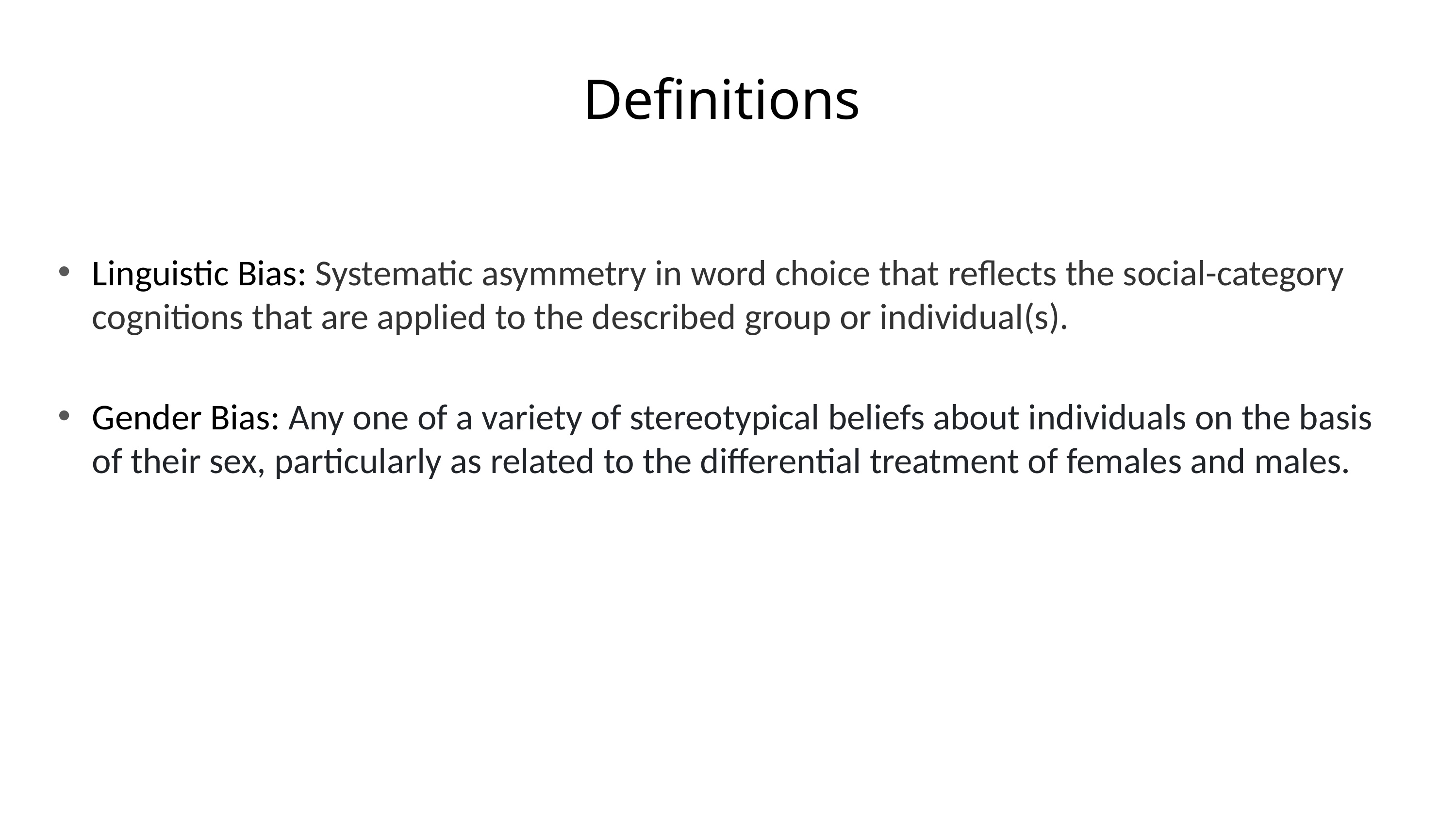

# Definitions
Linguistic Bias: Systematic asymmetry in word choice that reflects the social-category cognitions that are applied to the described group or individual(s).
Gender Bias: Any one of a variety of stereotypical beliefs about individuals on the basis of their sex, particularly as related to the differential treatment of females and males.

## Slide 5
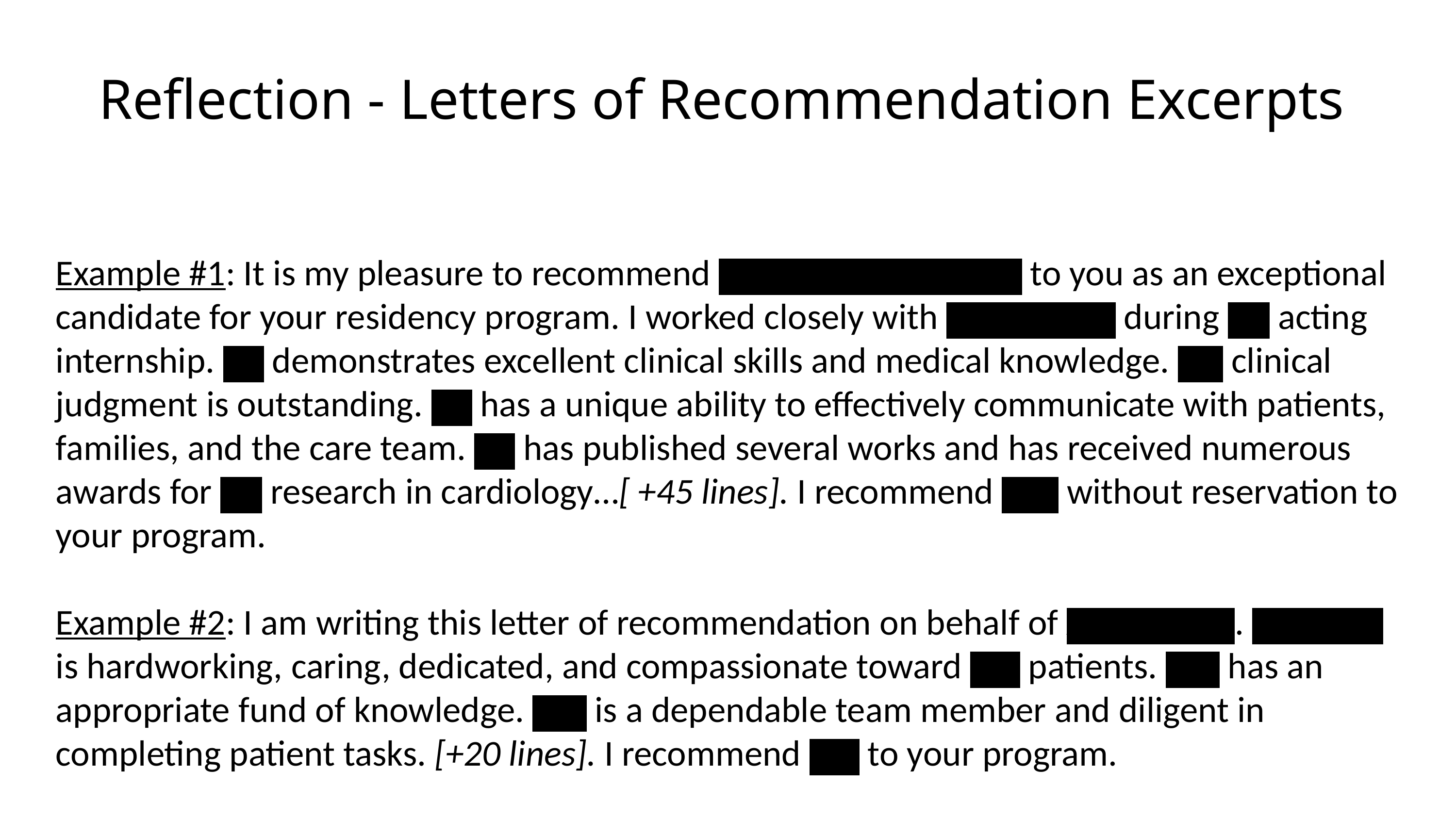

# Reflection - Letters of Recommendation Excerpts
Example #1: It is my pleasure to recommend Mr. Nicholas Holmes to you as an exceptional candidate for your residency program. I worked closely with Mr. Holmes during his acting internship. He demonstrates excellent clinical skills and medical knowledge. His clinical judgment is outstanding. He has a unique ability to effectively communicate with patients, families, and the care team. He has published several works and has received numerous awards for his research in cardiology…[ +45 lines]. I recommend him without reservation to your program.
Example #2: I am writing this letter of recommendation on behalf of Alyssa Sims. Ms. Sims is hardworking, caring, dedicated, and compassionate toward her patients. She has an appropriate fund of knowledge. She is a dependable team member and diligent in completing patient tasks. [+20 lines]. I recommend her to your program.

## Slide 6
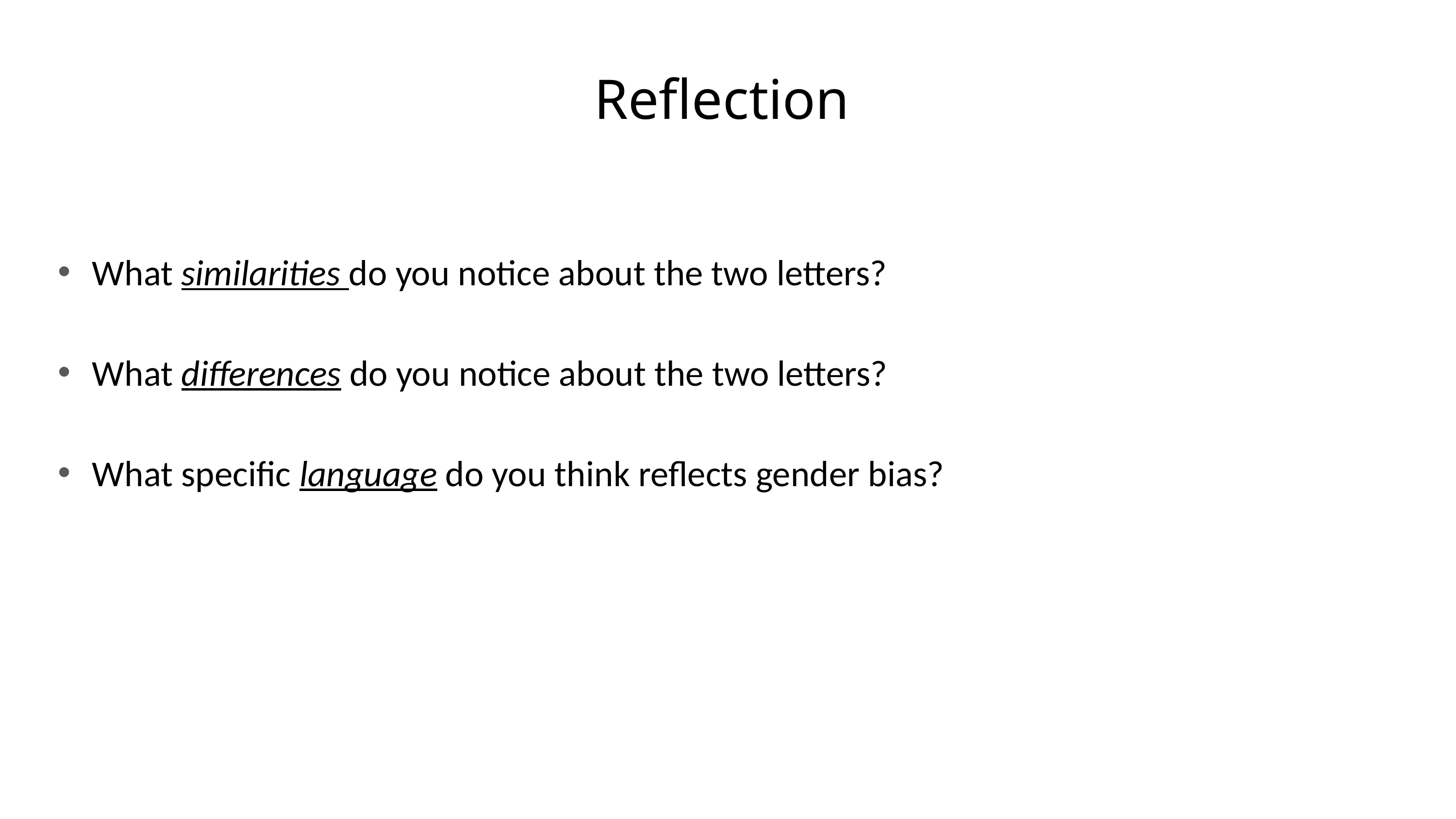

# Reflection
What similarities do you notice about the two letters?
What differences do you notice about the two letters?
What specific language do you think reflects gender bias?

## Slide 7
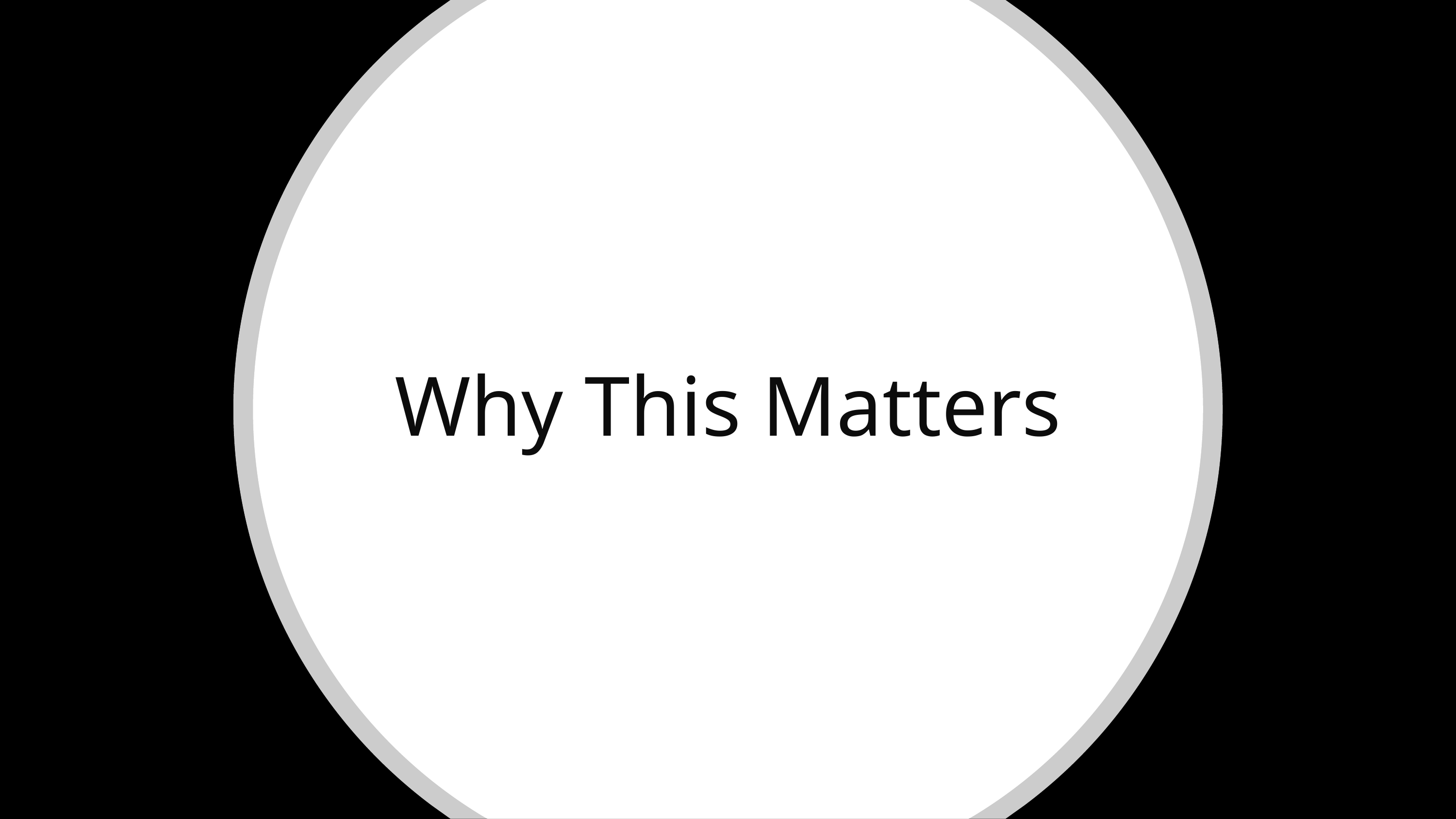

Why This Matters

## Slide 8
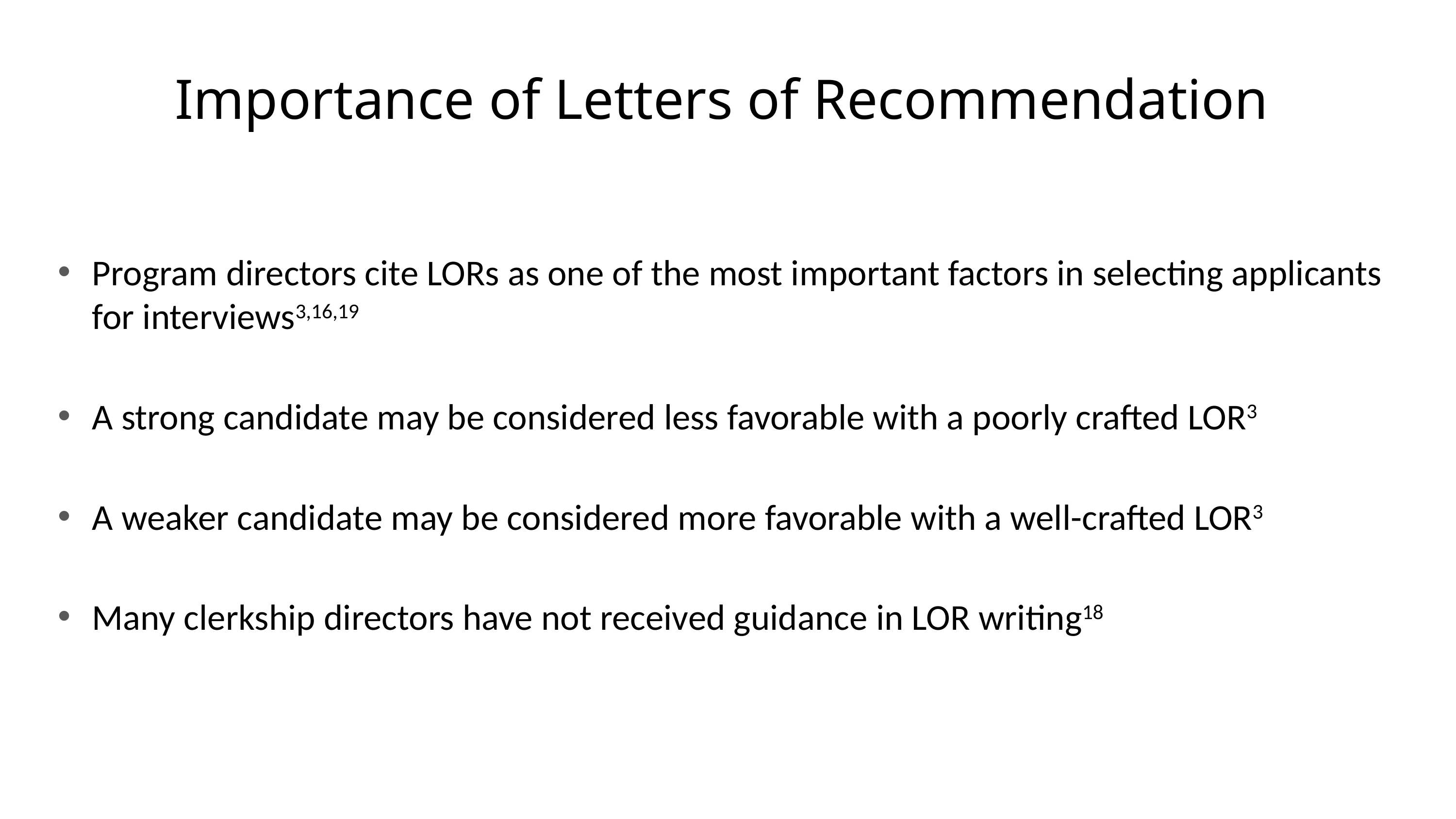

# Importance of Letters of Recommendation
Program directors cite LORs as one of the most important factors in selecting applicants for interviews3,16,19
A strong candidate may be considered less favorable with a poorly crafted LOR3
A weaker candidate may be considered more favorable with a well-crafted LOR3
Many clerkship directors have not received guidance in LOR writing18

## Slide 9
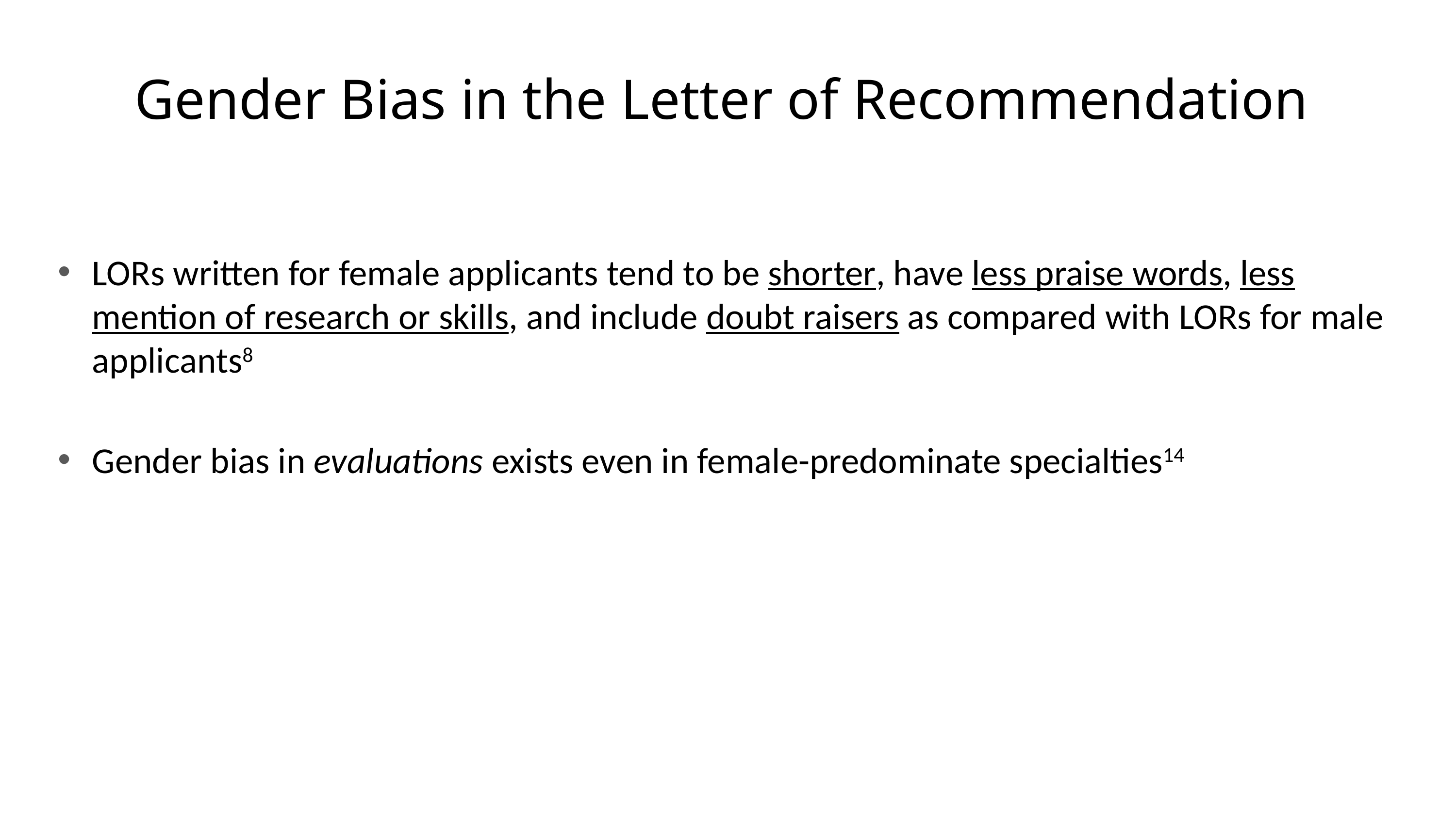

# Gender Bias in the Letter of Recommendation
LORs written for female applicants tend to be shorter, have less praise words, less mention of research or skills, and include doubt raisers as compared with LORs for male applicants8
Gender bias in evaluations exists even in female-predominate specialties14

## Slide 10
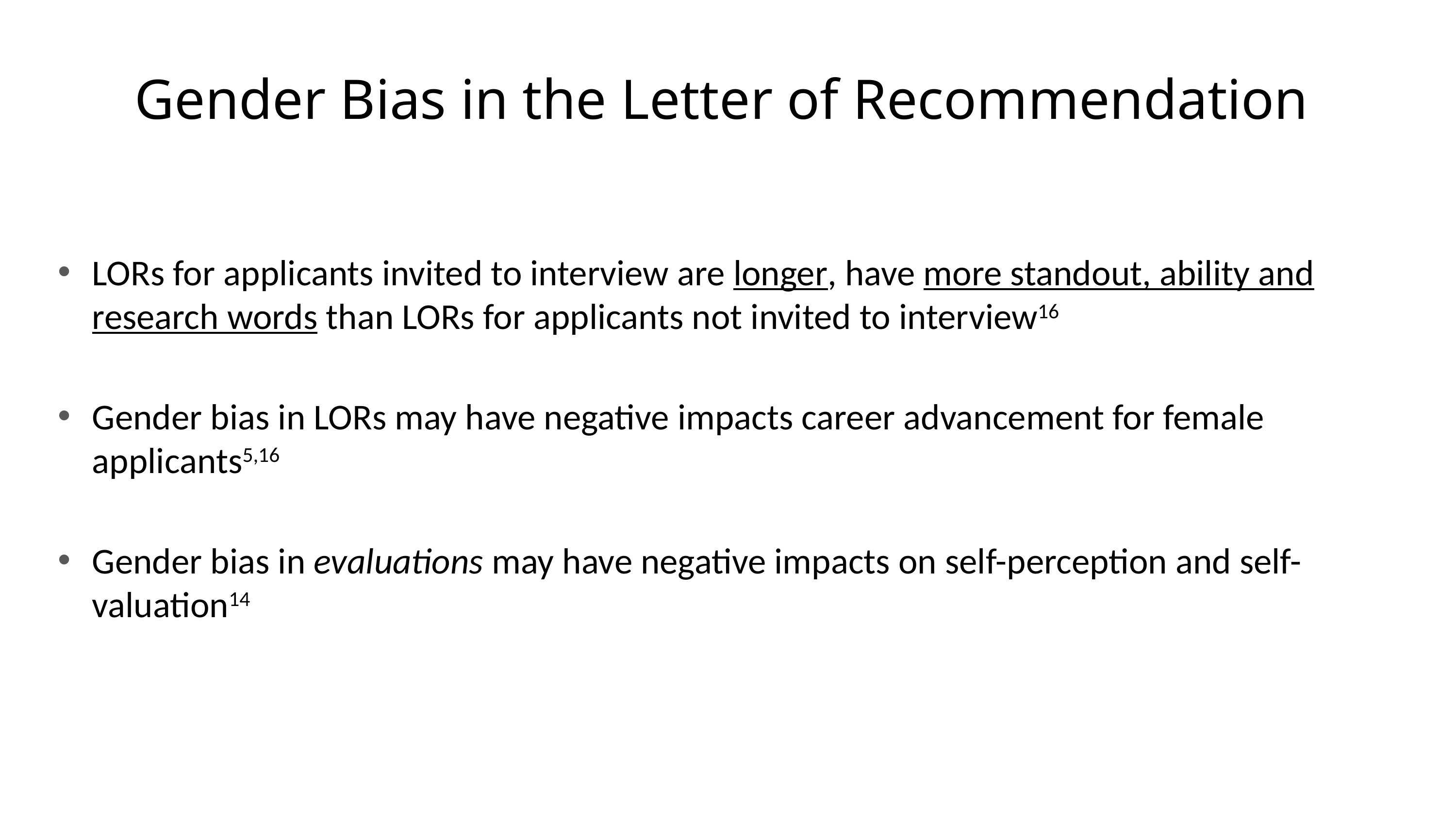

# Gender Bias in the Letter of Recommendation
LORs for applicants invited to interview are longer, have more standout, ability and research words than LORs for applicants not invited to interview16
Gender bias in LORs may have negative impacts career advancement for female applicants5,16
Gender bias in evaluations may have negative impacts on self-perception and self-valuation14

## Slide 11
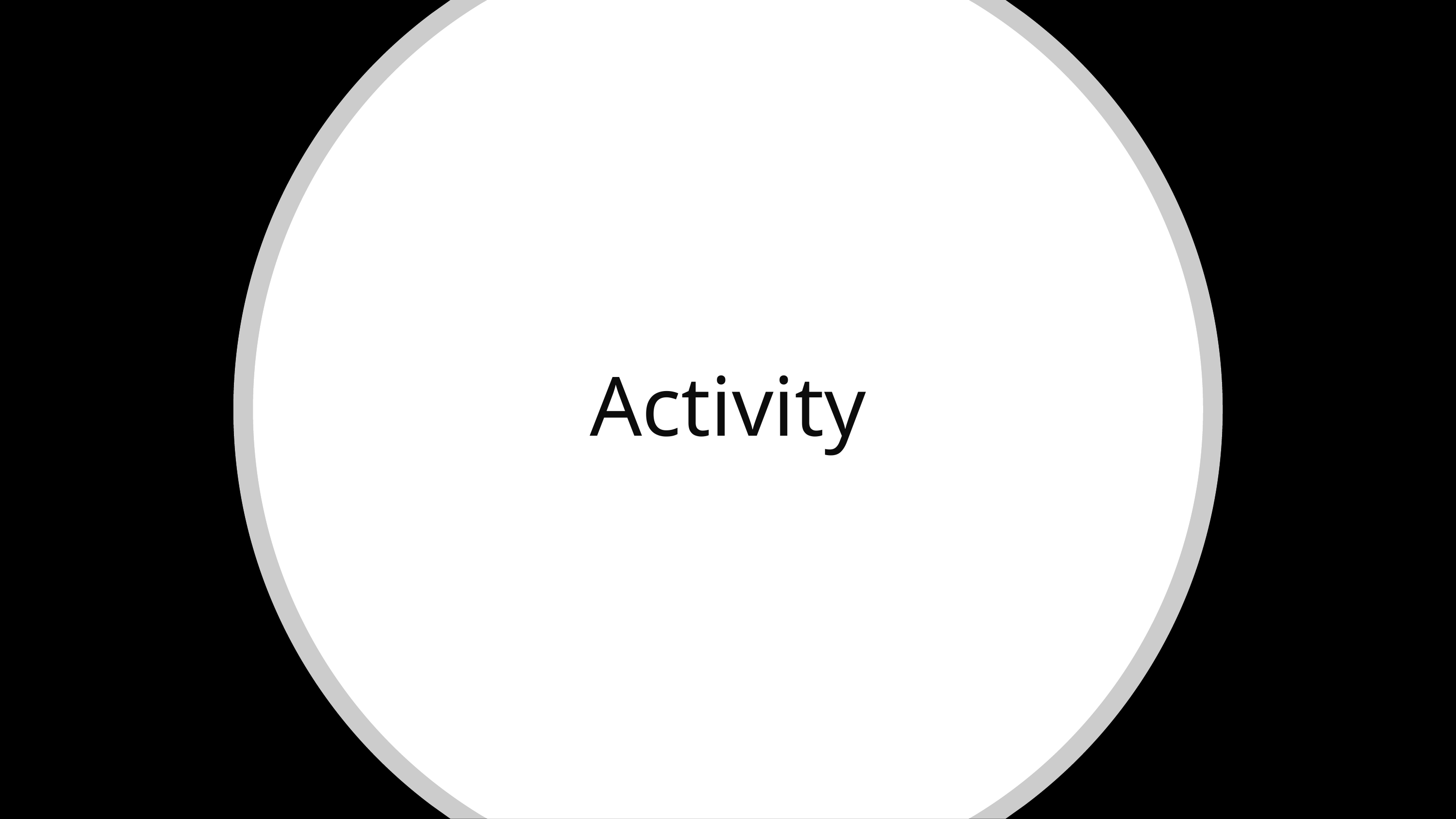

Activity

## Slide 12
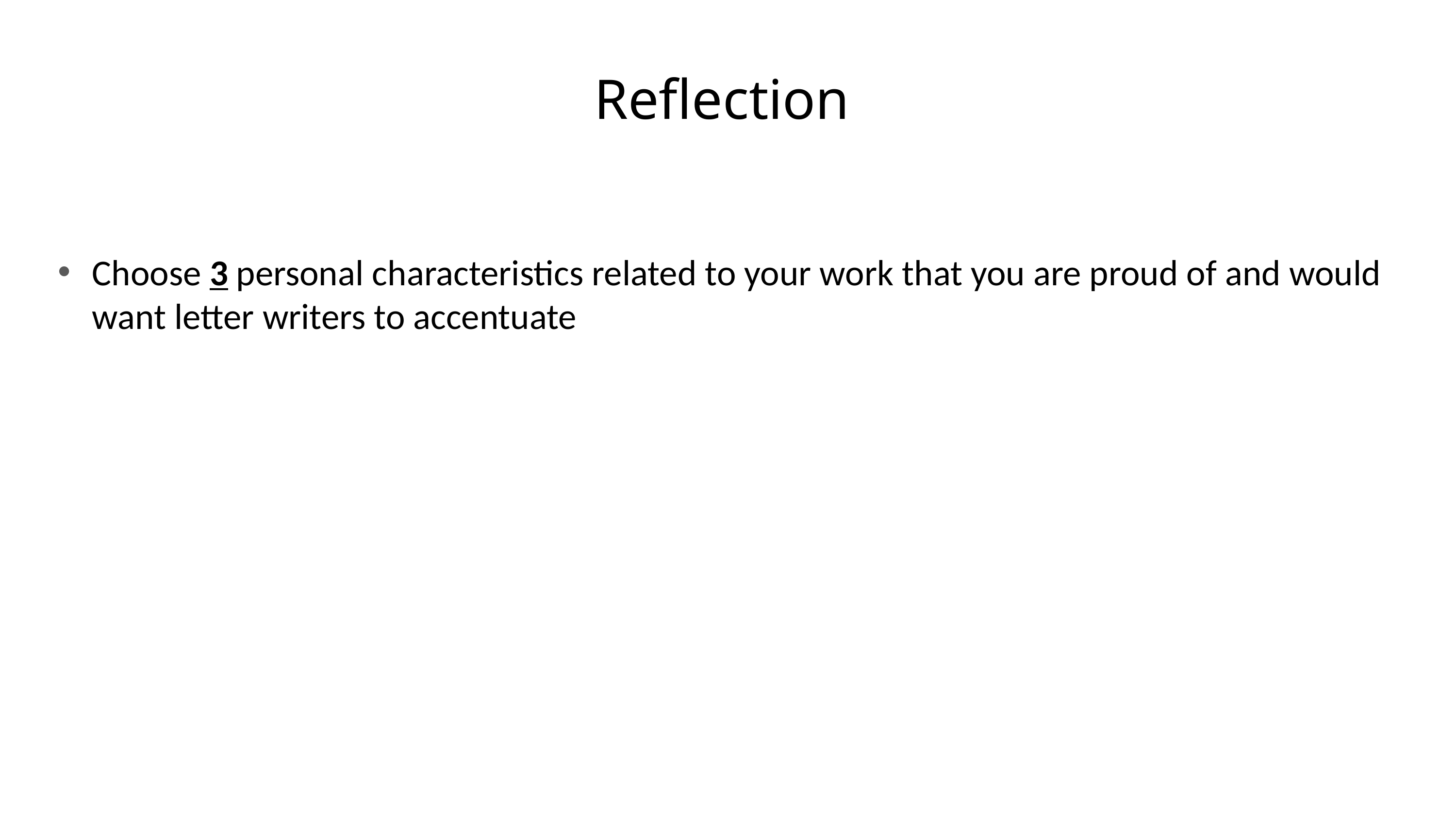

# Reflection
Choose 3 personal characteristics related to your work that you are proud of and would want letter writers to accentuate

## Slide 13
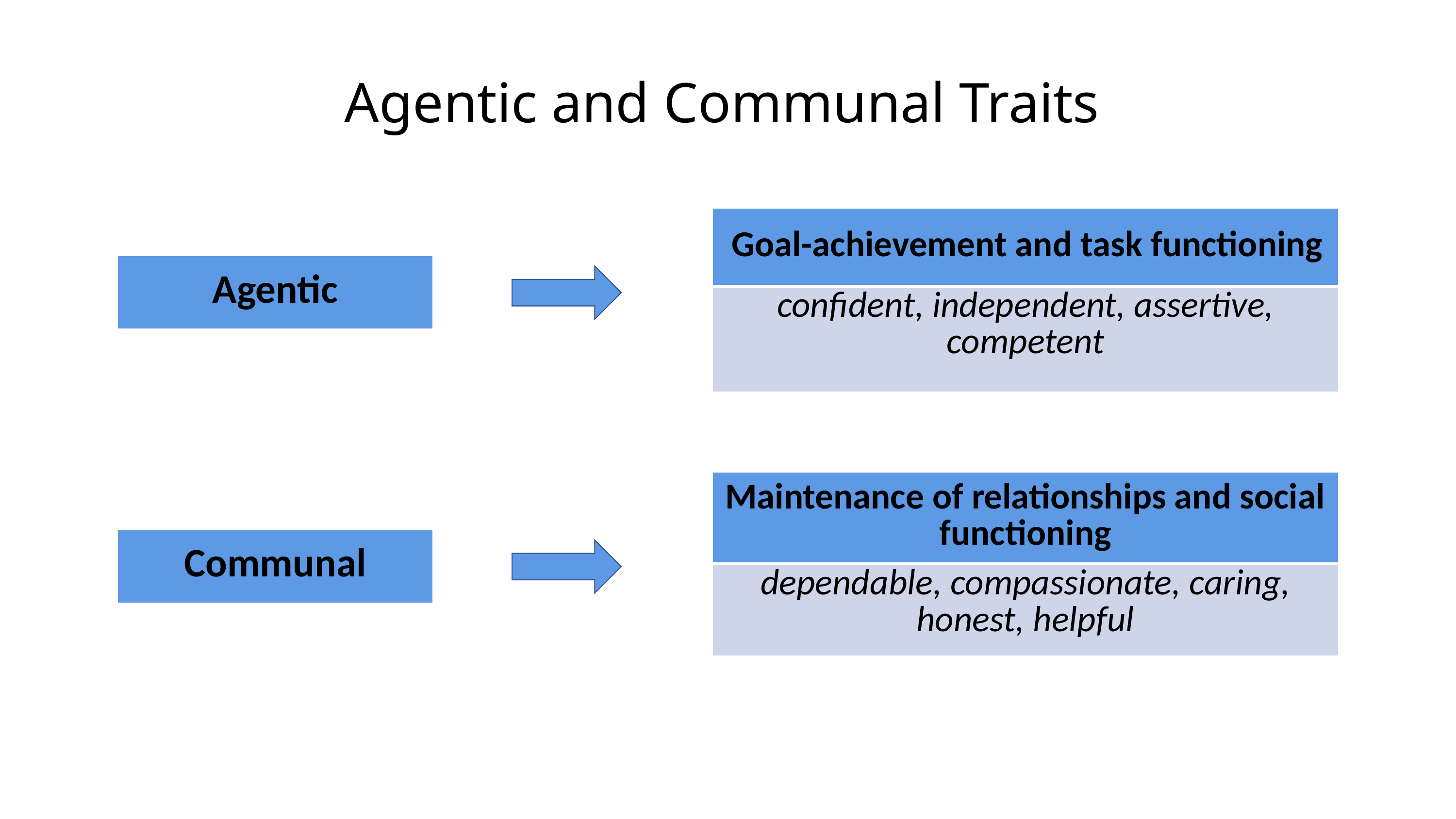

# Agentic and Communal Traits
| Goal-achievement and task functioning |
| --- |
| confident, independent, assertive, competent |
| Agentic |
| --- |
| Maintenance of relationships and social functioning |
| --- |
| dependable, compassionate, caring, honest, helpful |
| Communal |
| --- |

## Slide 14
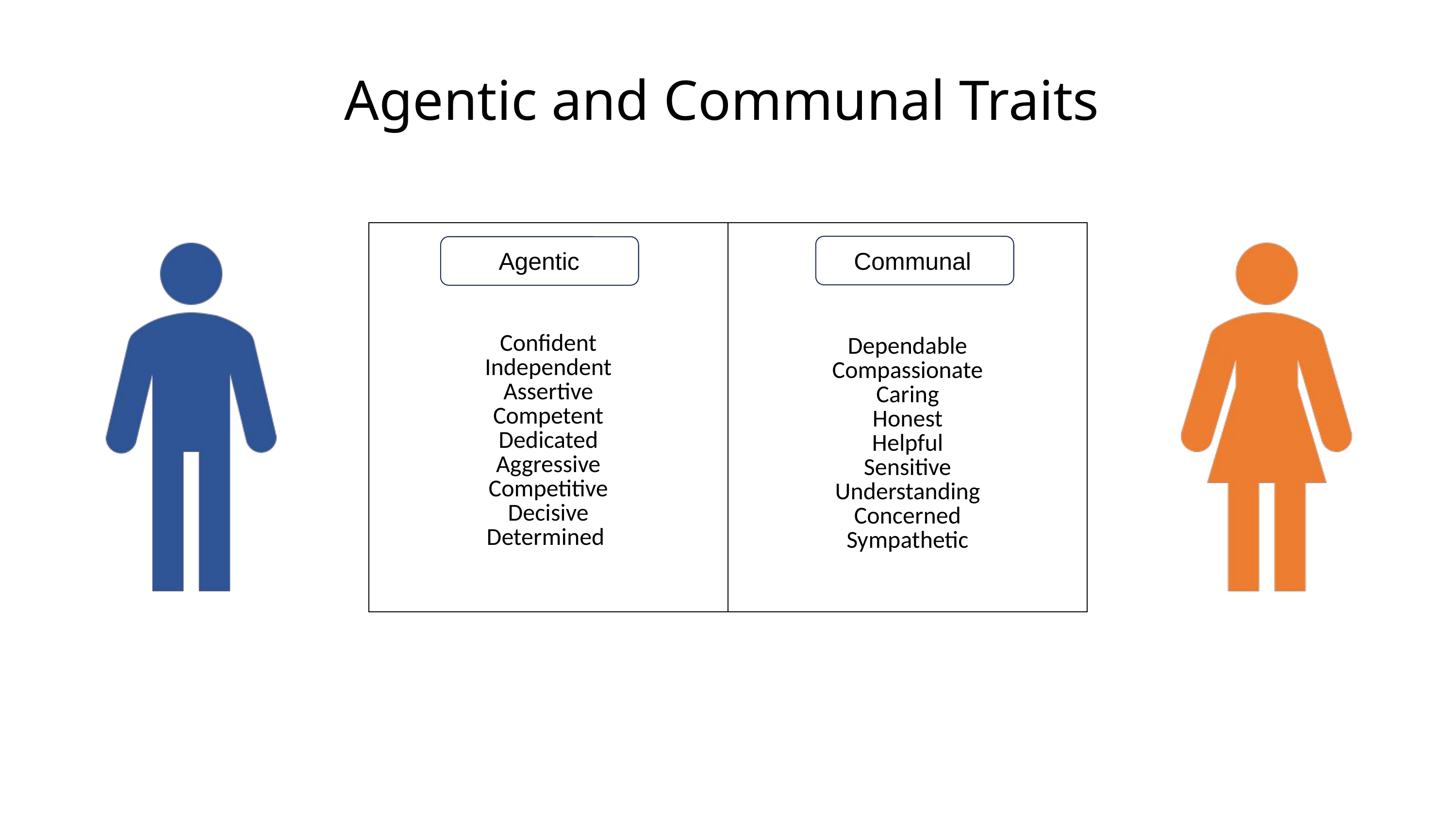

# Agentic and Communal Traits
| Confident Independent Assertive Competent Dedicated Aggressive Competitive Decisive Determined | Dependable Compassionate Caring Honest Helpful Sensitive Understanding Concerned Sympathetic |
| --- | --- |
Communal
Agentic

## Slide 15
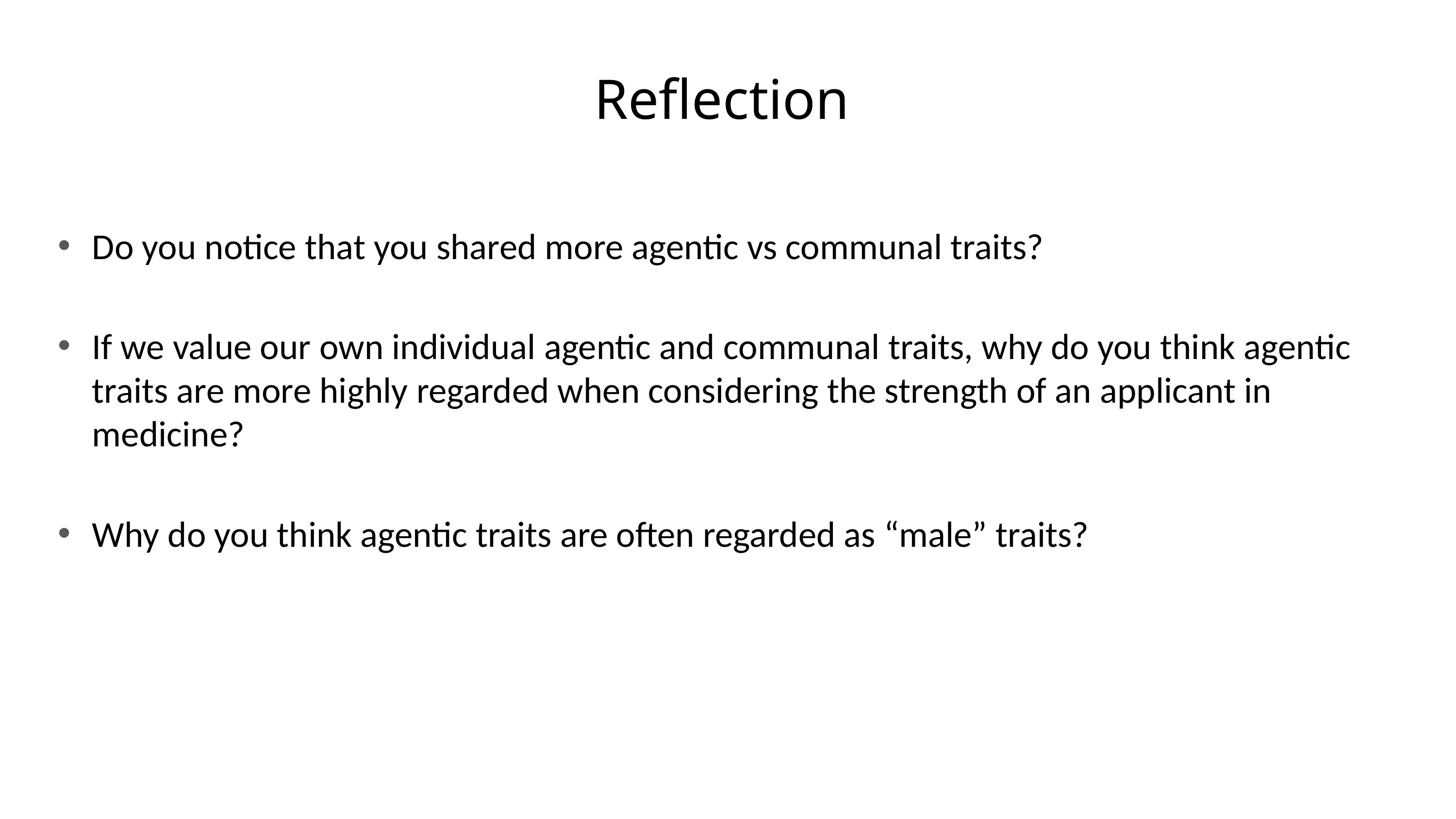

# Reflection
Do you notice that you shared more agentic vs communal traits?
If we value our own individual agentic and communal traits, why do you think agentic traits are more highly regarded when considering the strength of an applicant in medicine?
Why do you think agentic traits are often regarded as “male” traits?

## Slide 16
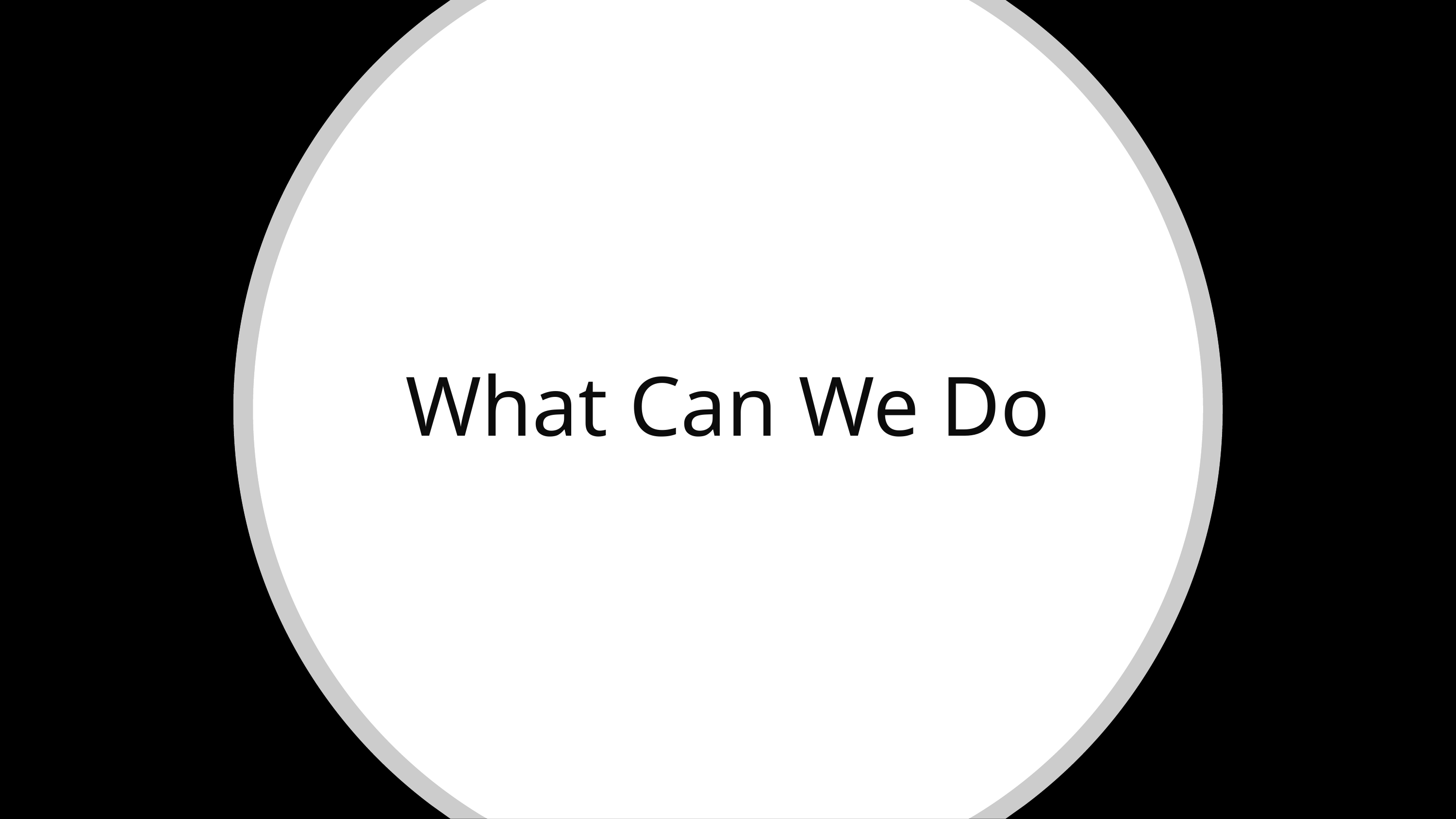

What Can We Do

## Slide 17
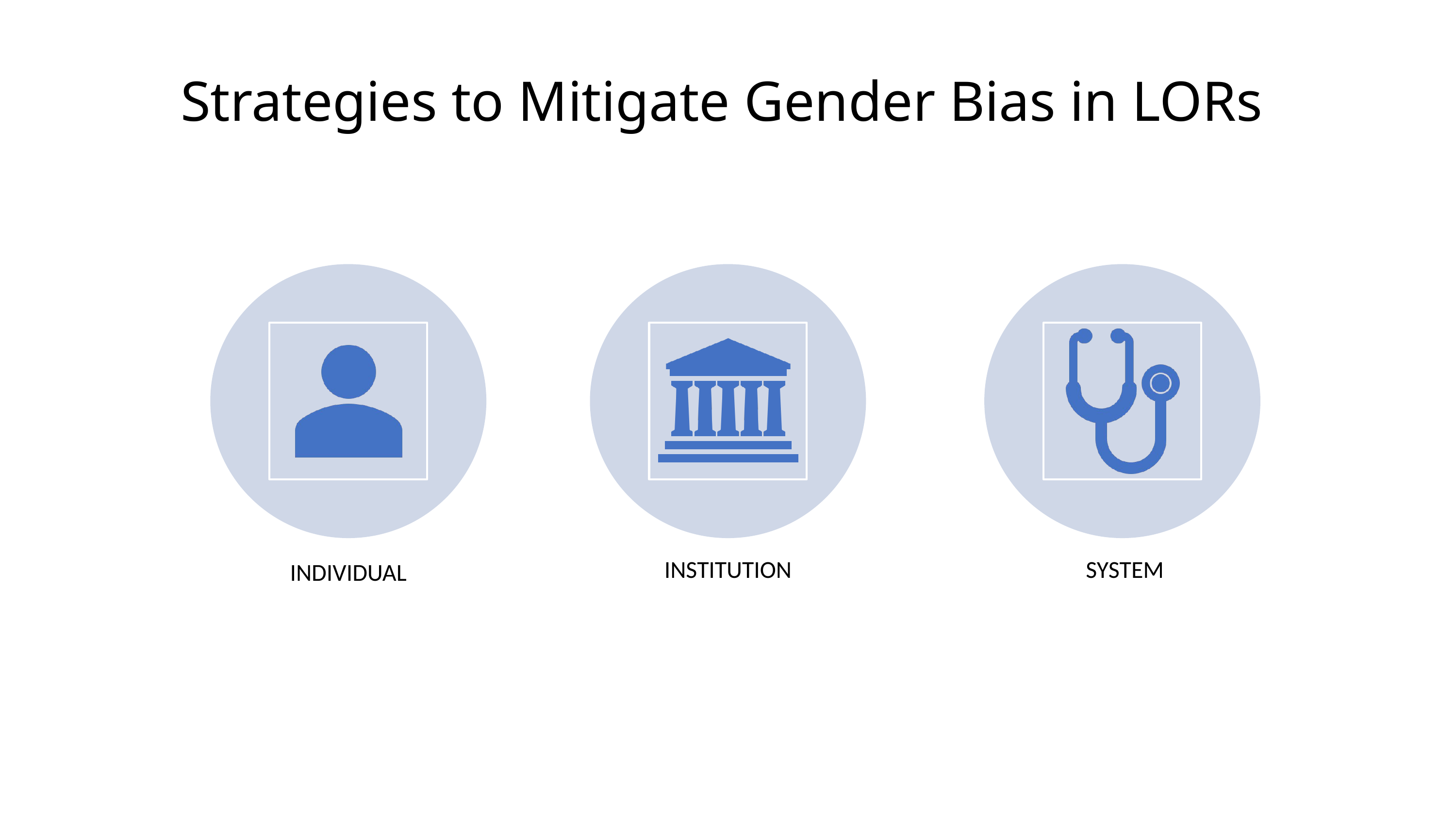

# Strategies to Mitigate Gender Bias in LORs
INSTITUTION
SYSTEM
INDIVIDUAL

## Slide 18
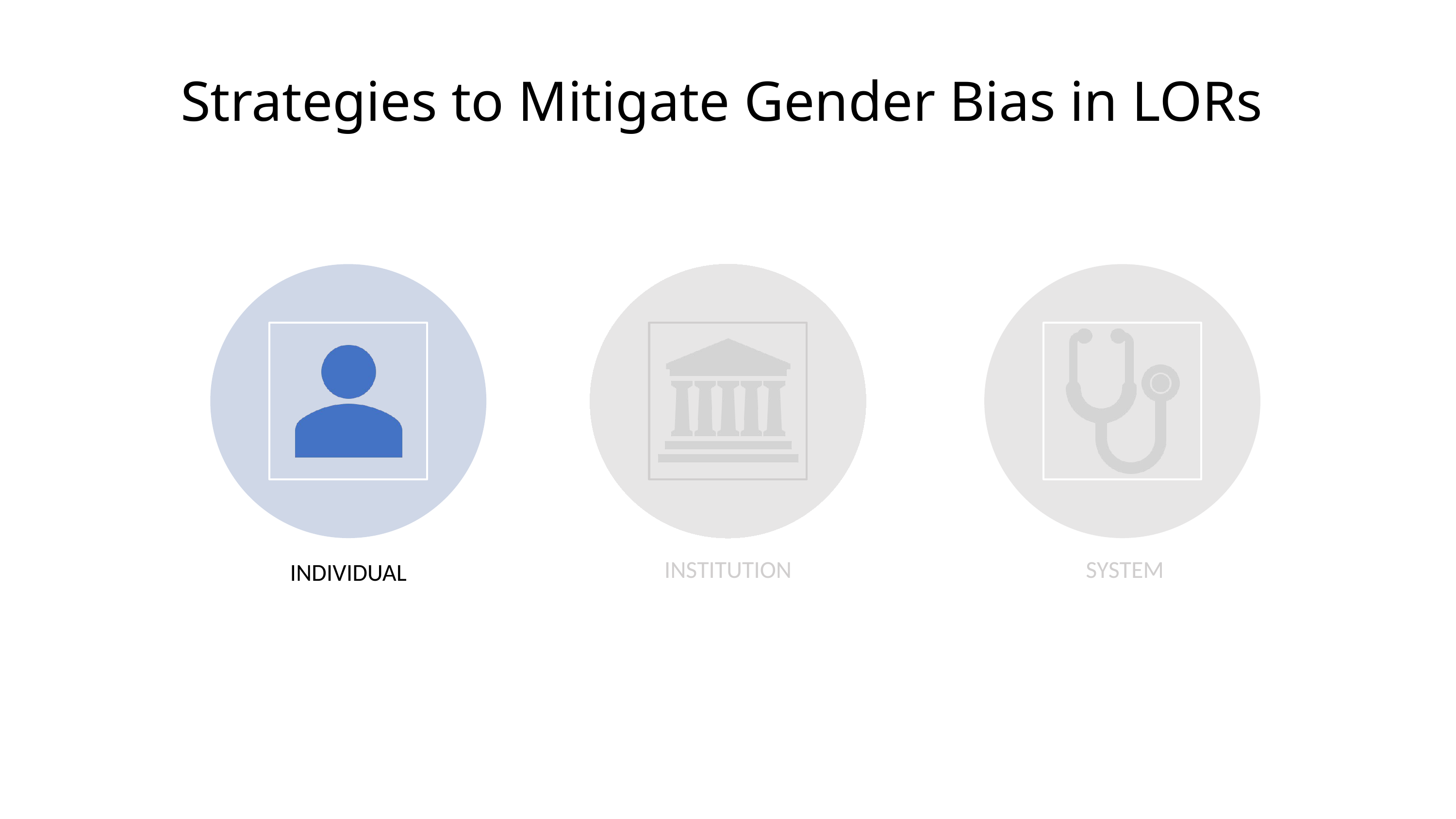

# Strategies to Mitigate Gender Bias in LORs
INSTITUTION
SYSTEM
INDIVIDUAL

## Slide 19
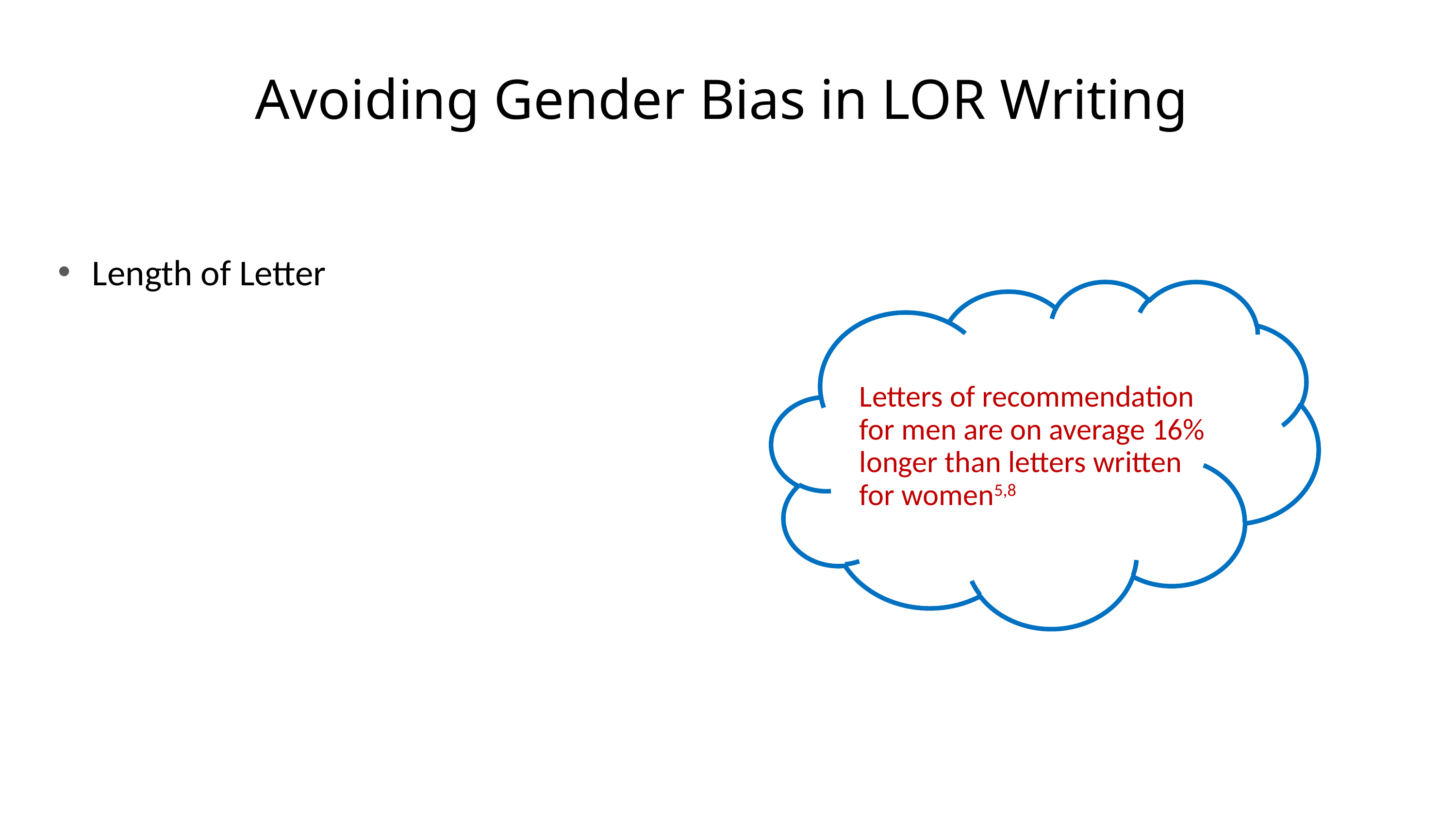

# Avoiding Gender Bias in LOR Writing
Length of Letter
Letters of recommendation for men are on average 16% longer than letters written for women5,8

## Slide 20
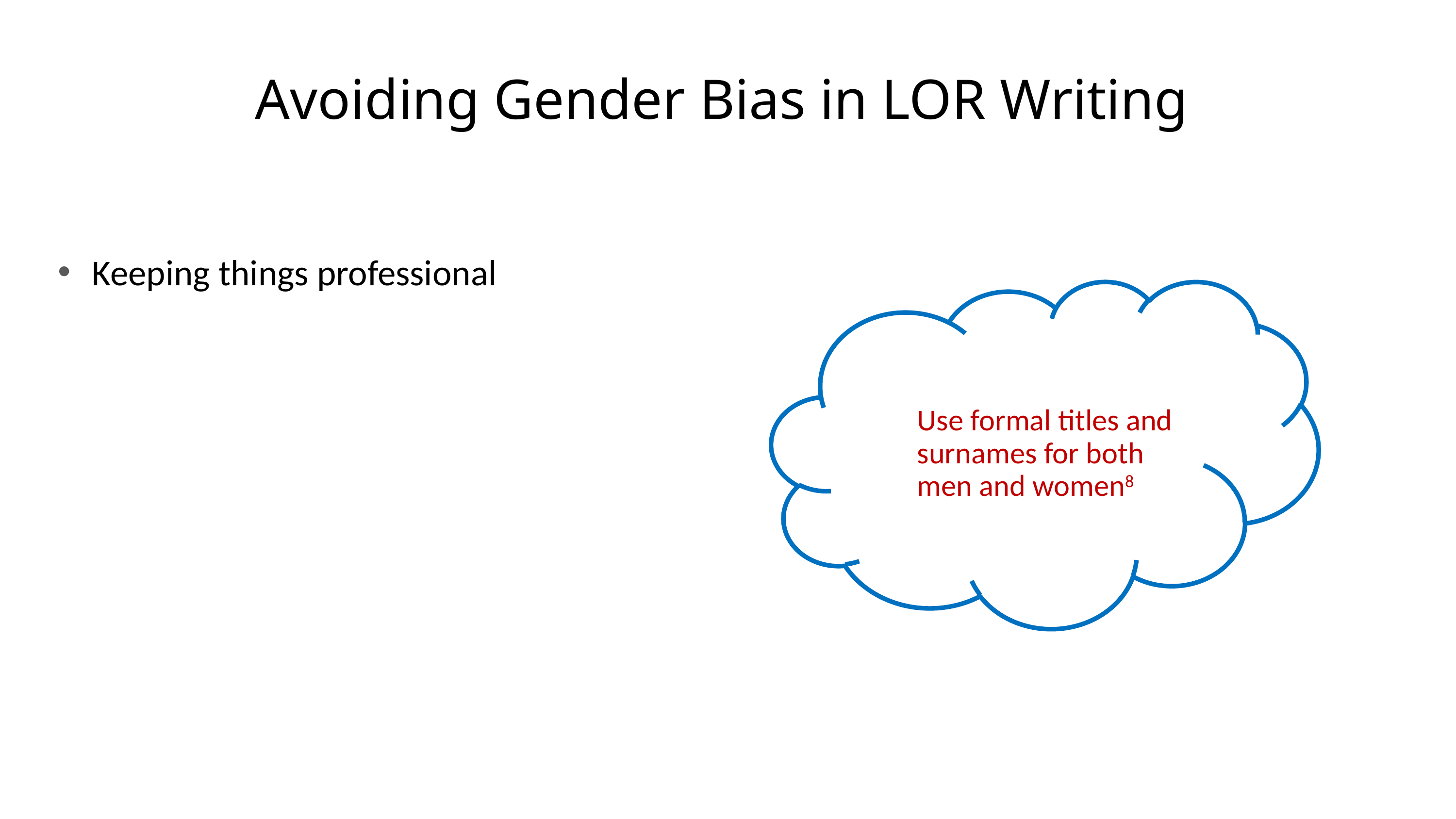

# Avoiding Gender Bias in LOR Writing
Keeping things professional
Use formal titles and surnames for both
men and women8

## Slide 21
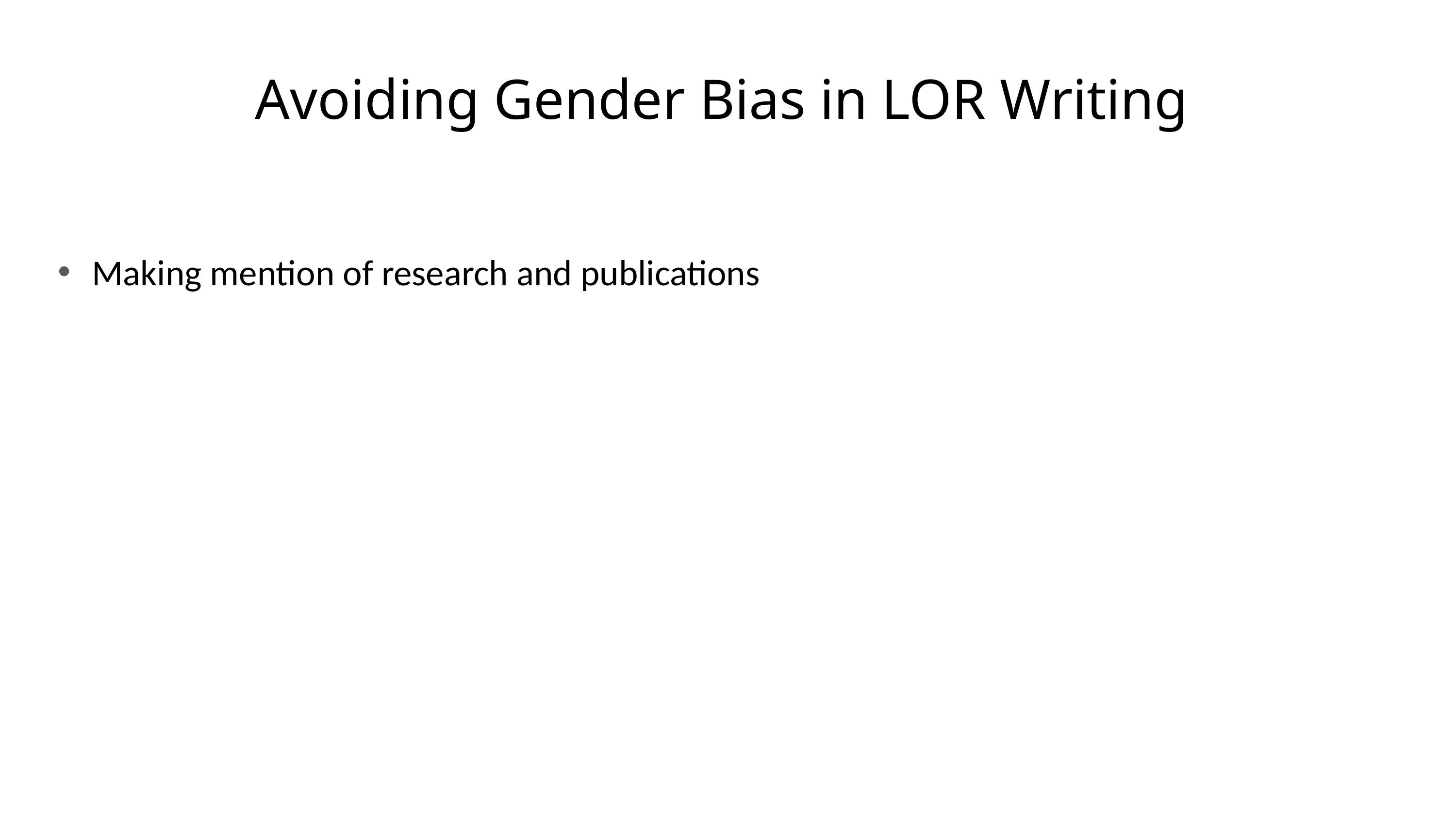

# Avoiding Gender Bias in LOR Writing
Making mention of research and publications

## Slide 22
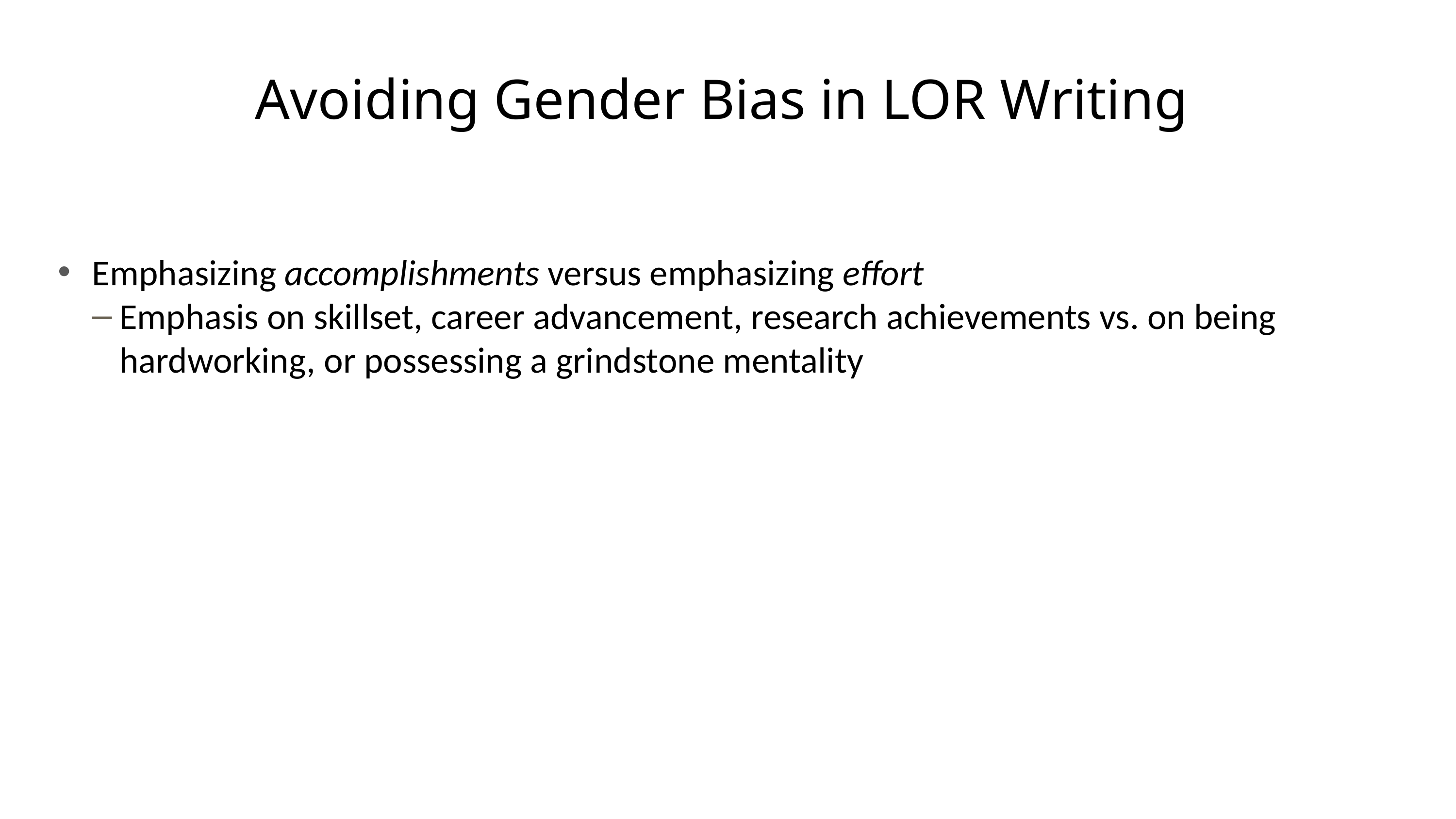

# Avoiding Gender Bias in LOR Writing
Emphasizing accomplishments versus emphasizing effort
Emphasis on skillset, career advancement, research achievements vs. on being hardworking, or possessing a grindstone mentality

## Slide 23
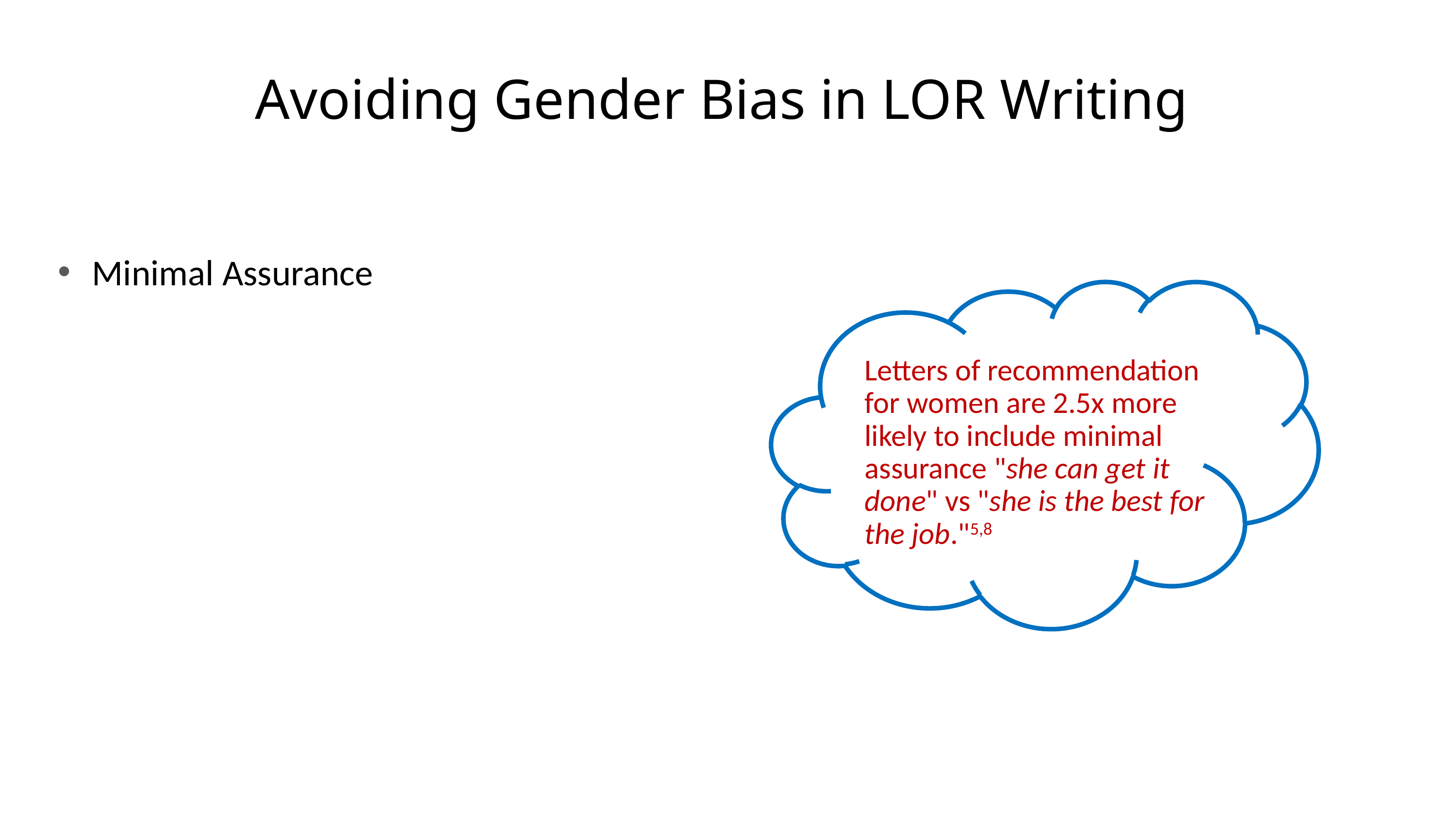

# Avoiding Gender Bias in LOR Writing
Minimal Assurance
Letters of recommendation for women are 2.5x more likely to include minimal assurance "she can get it done" vs "she is the best for the job."5,8

## Slide 24
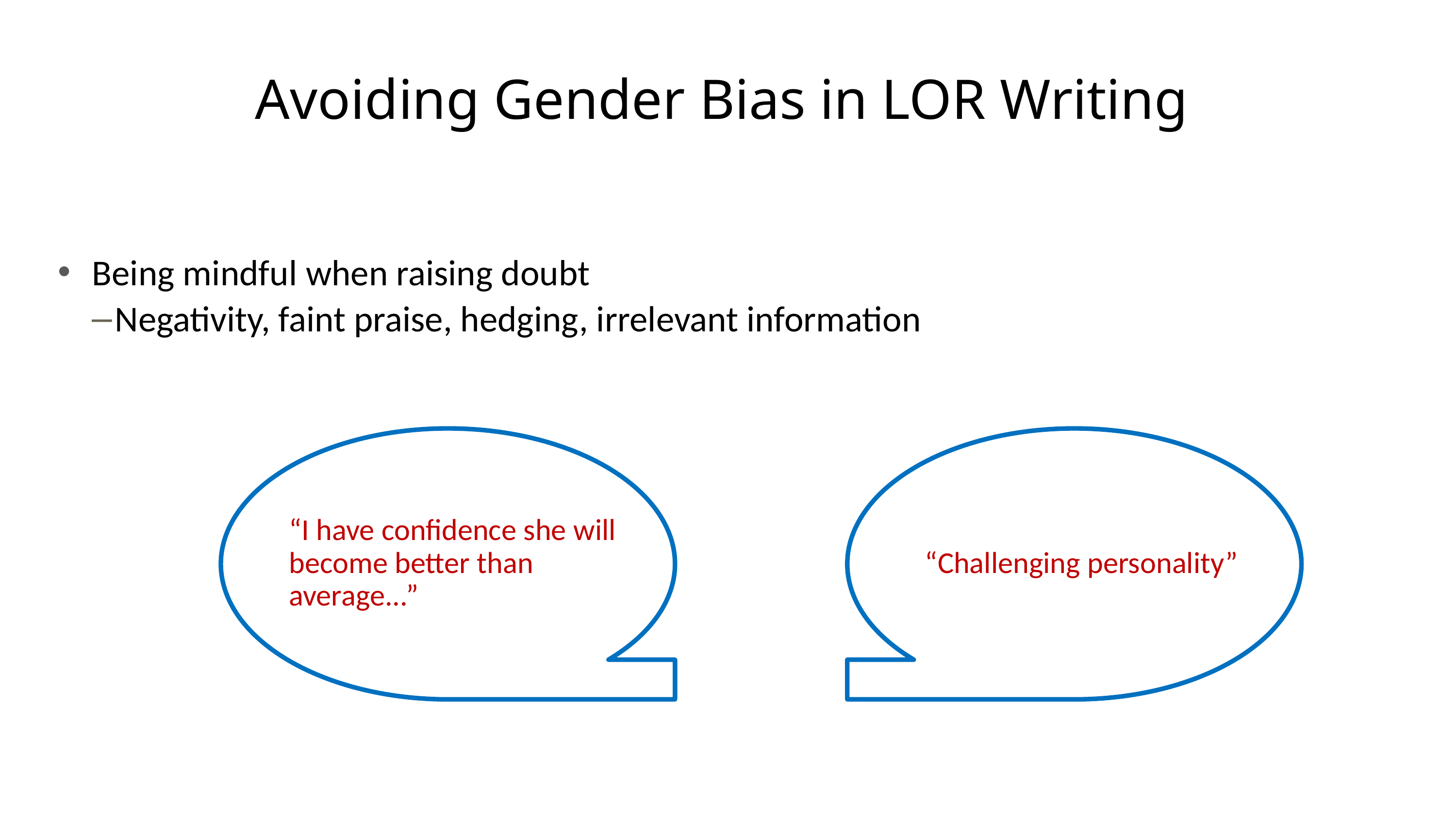

# Avoiding Gender Bias in LOR Writing
Being mindful when raising doubt
Negativity, faint praise, hedging, irrelevant information
“I have confidence she will become better than average...”
“Challenging personality”

## Slide 25
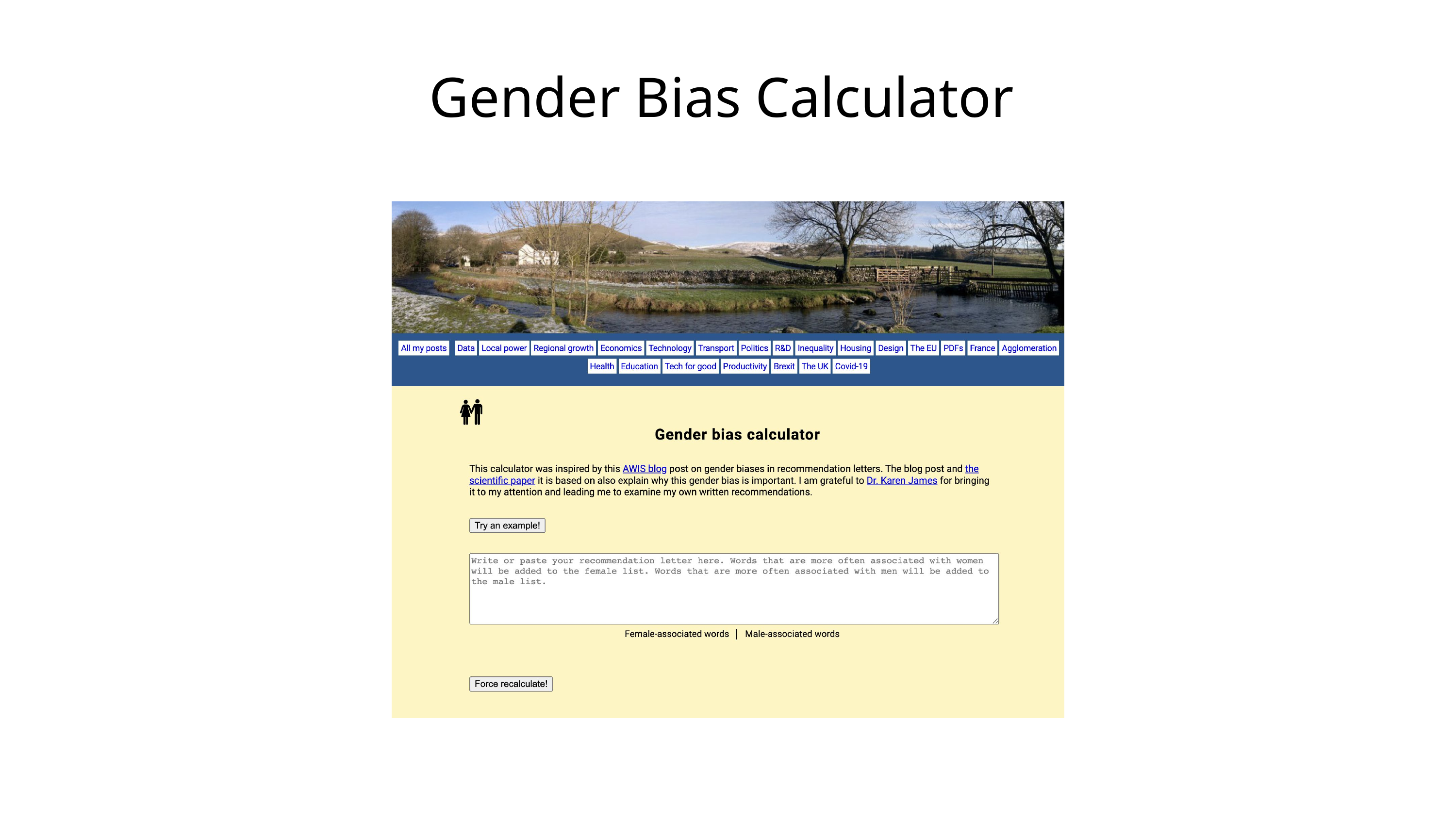

# Gender Bias Calculator

## Slide 26
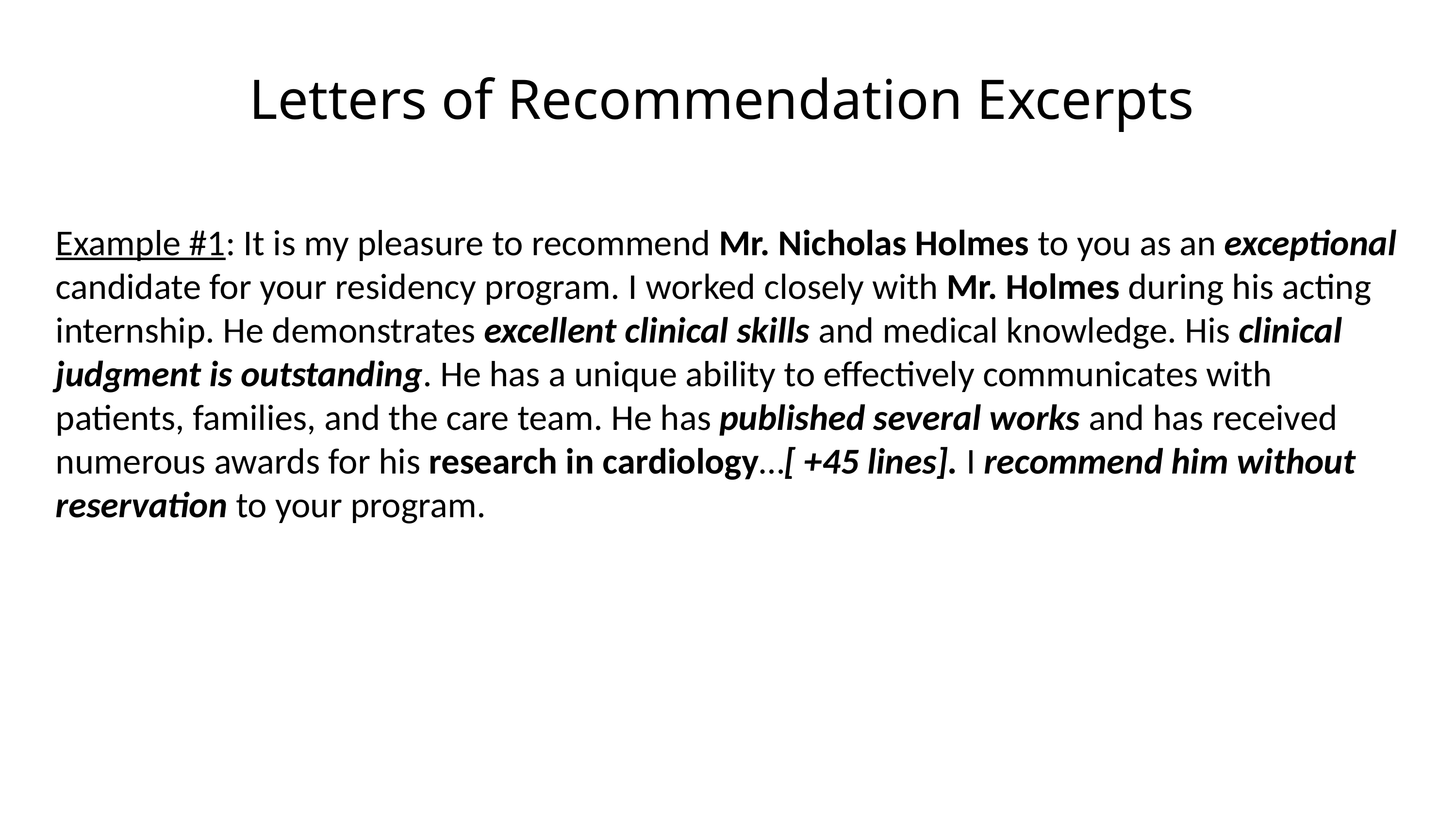

# Letters of Recommendation Excerpts
Example #1: It is my pleasure to recommend Mr. Nicholas Holmes to you as an exceptional candidate for your residency program. I worked closely with Mr. Holmes during his acting internship. He demonstrates excellent clinical skills and medical knowledge. His clinical judgment is outstanding. He has a unique ability to effectively communicates with patients, families, and the care team. He has published several works and has received numerous awards for his research in cardiology…[ +45 lines]. I recommend him without reservation to your program.

## Slide 27
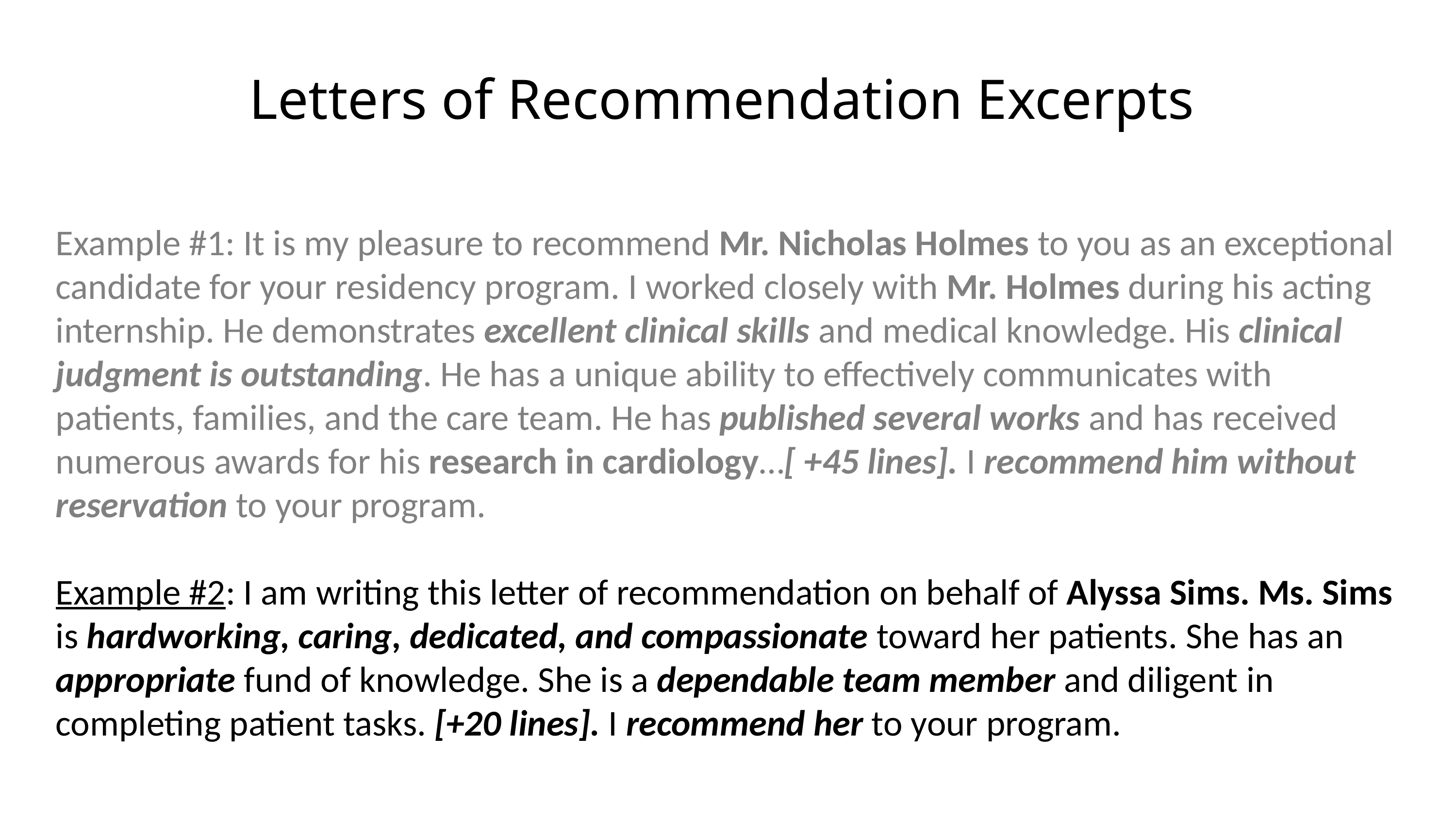

# Letters of Recommendation Excerpts
Example #1: It is my pleasure to recommend Mr. Nicholas Holmes to you as an exceptional candidate for your residency program. I worked closely with Mr. Holmes during his acting internship. He demonstrates excellent clinical skills and medical knowledge. His clinical judgment is outstanding. He has a unique ability to effectively communicates with patients, families, and the care team. He has published several works and has received numerous awards for his research in cardiology…[ +45 lines]. I recommend him without reservation to your program.
Example #2: I am writing this letter of recommendation on behalf of Alyssa Sims. Ms. Sims is hardworking, caring, dedicated, and compassionate toward her patients. She has an appropriate fund of knowledge. She is a dependable team member and diligent in completing patient tasks. [+20 lines]. I recommend her to your program.

## Slide 28
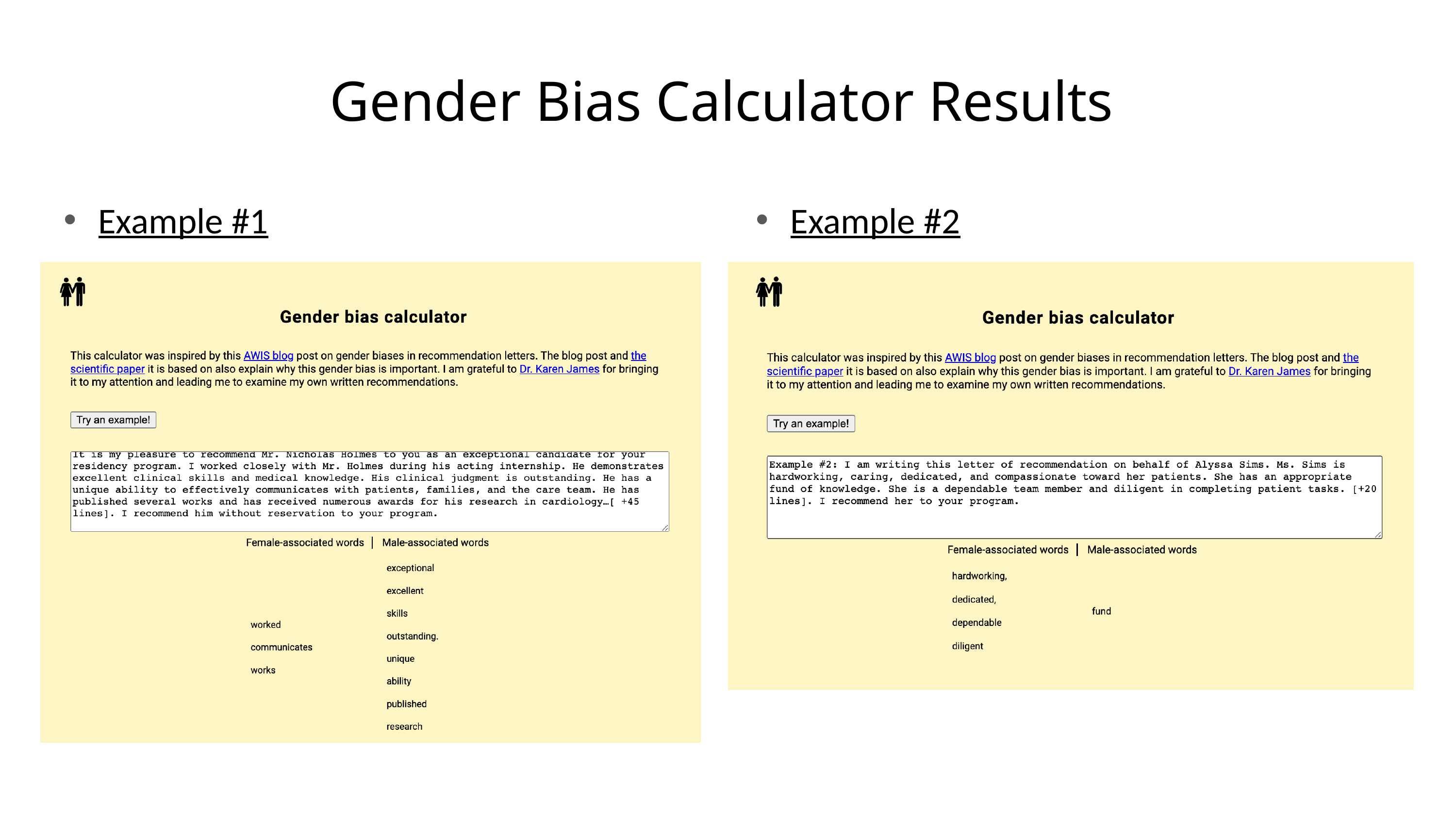

# Gender Bias Calculator Results
Example #1
Example #2

## Slide 29
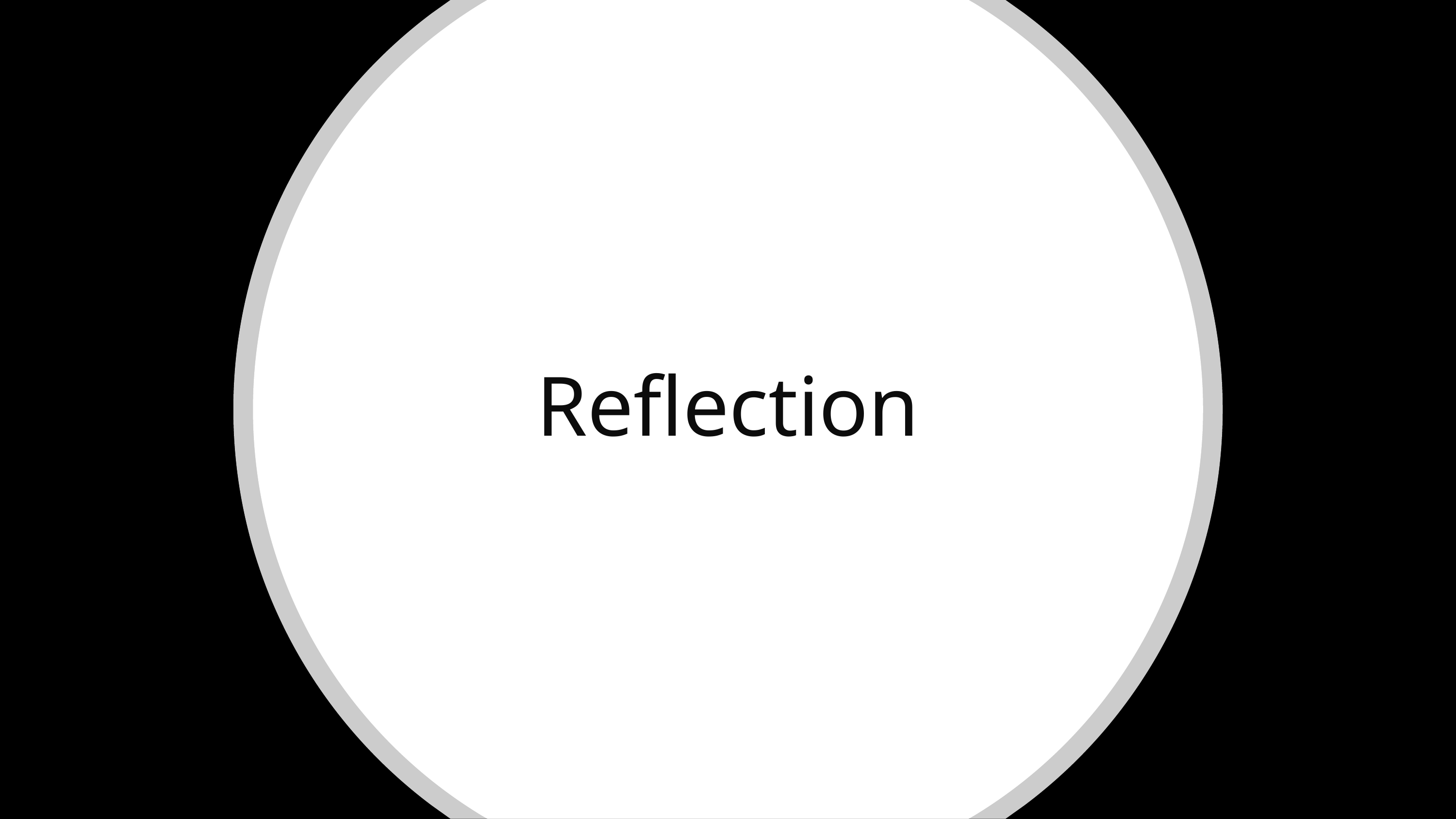

Reflection

## Slide 30
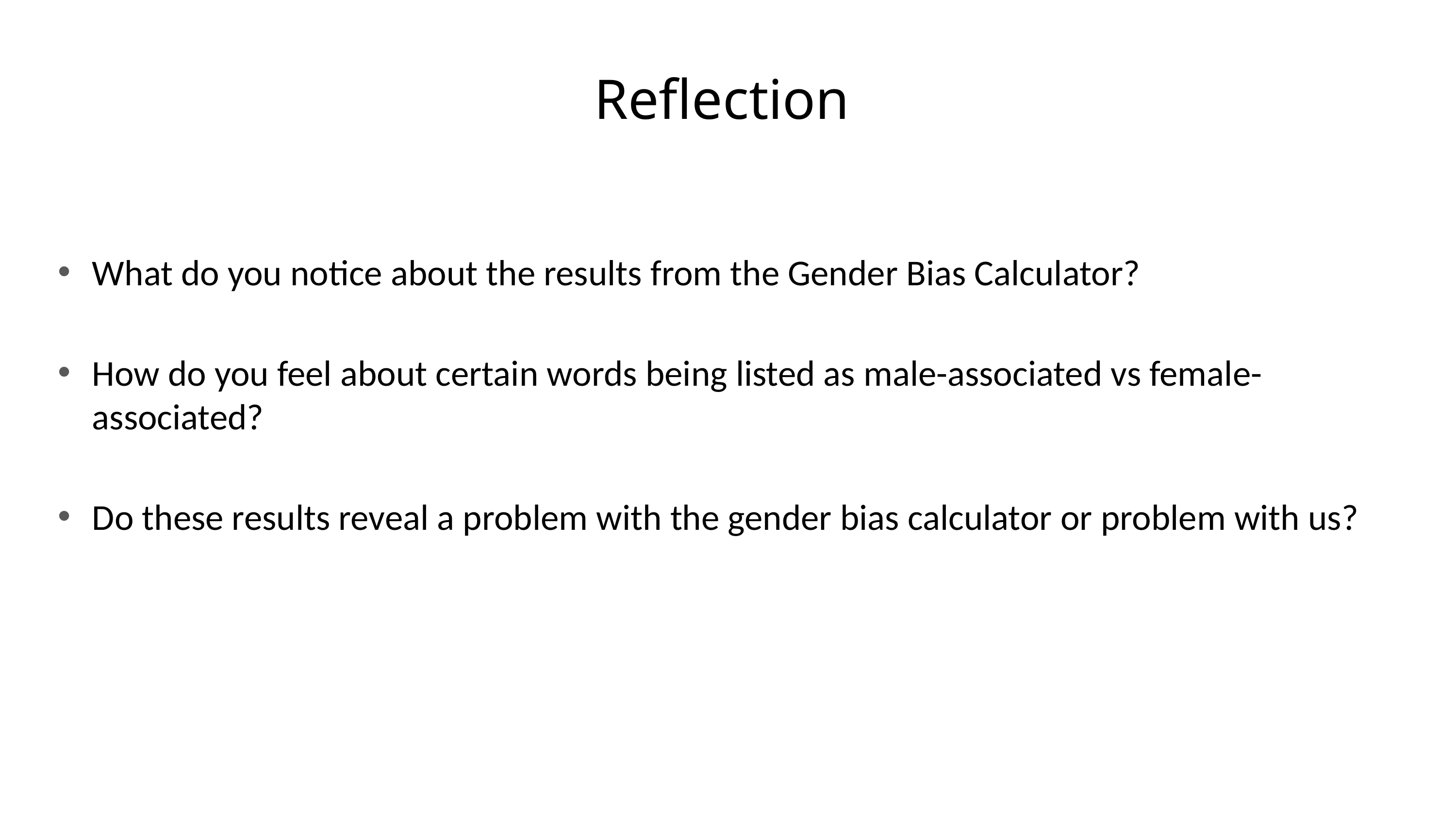

# Reflection
What do you notice about the results from the Gender Bias Calculator?
How do you feel about certain words being listed as male-associated vs female-associated?
Do these results reveal a problem with the gender bias calculator or problem with us?

## Slide 31
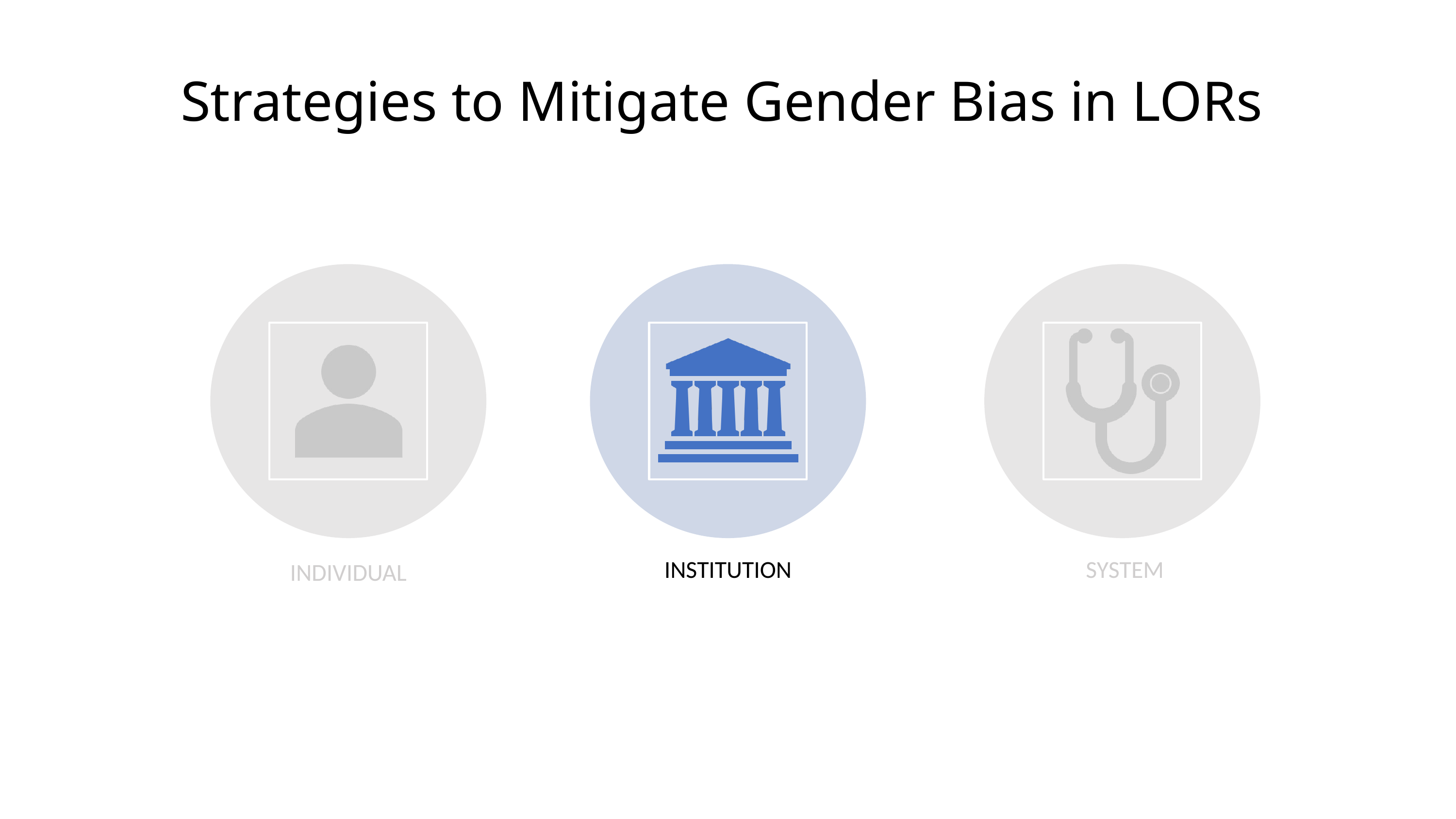

# Strategies to Mitigate Gender Bias in LORs
INSTITUTION
SYSTEM
INDIVIDUAL

## Slide 32
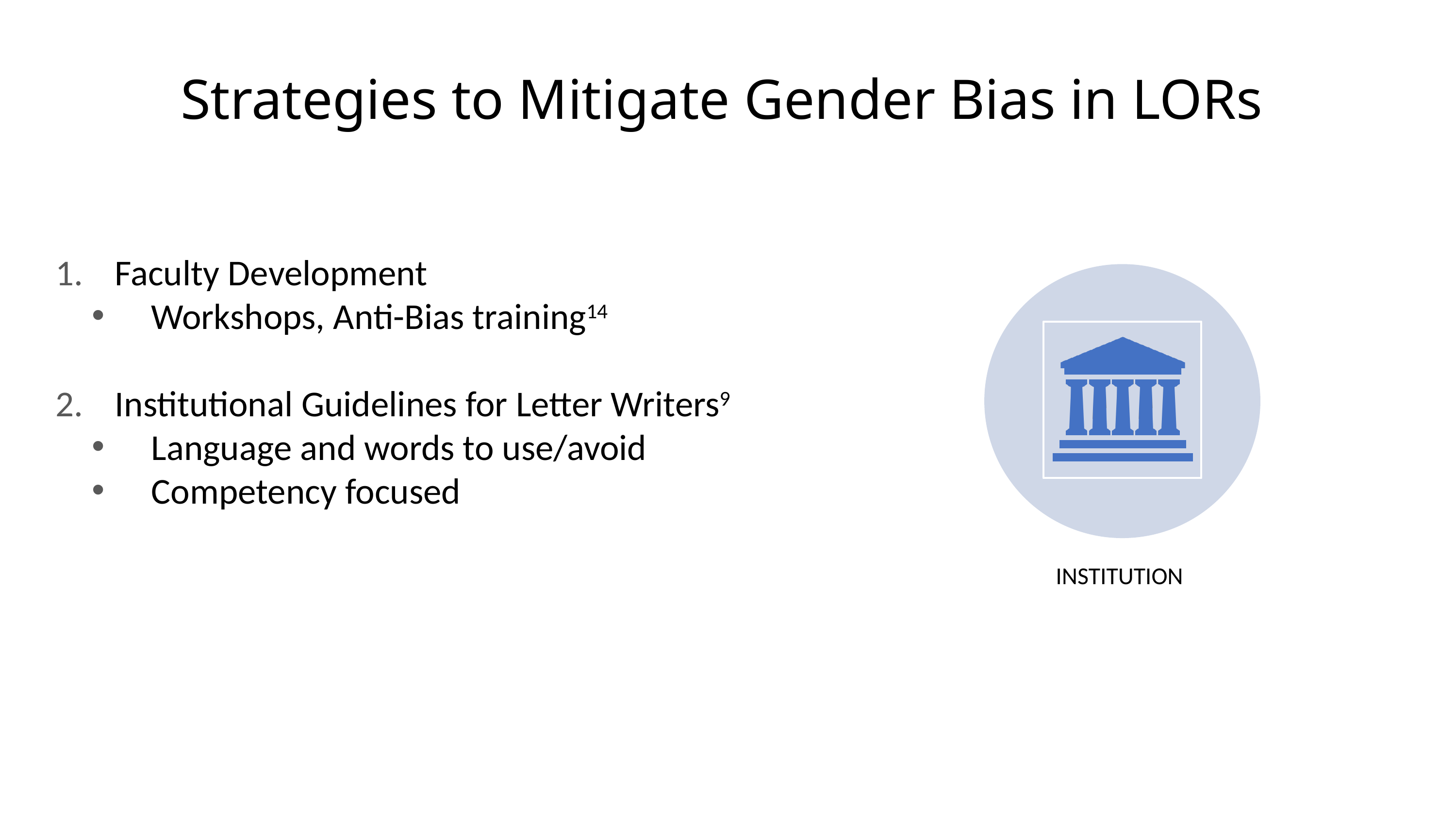

# Strategies to Mitigate Gender Bias in LORs
Faculty Development
Workshops, Anti-Bias training14
Institutional Guidelines for Letter Writers9
Language and words to use/avoid
Competency focused
INSTITUTION

## Slide 33
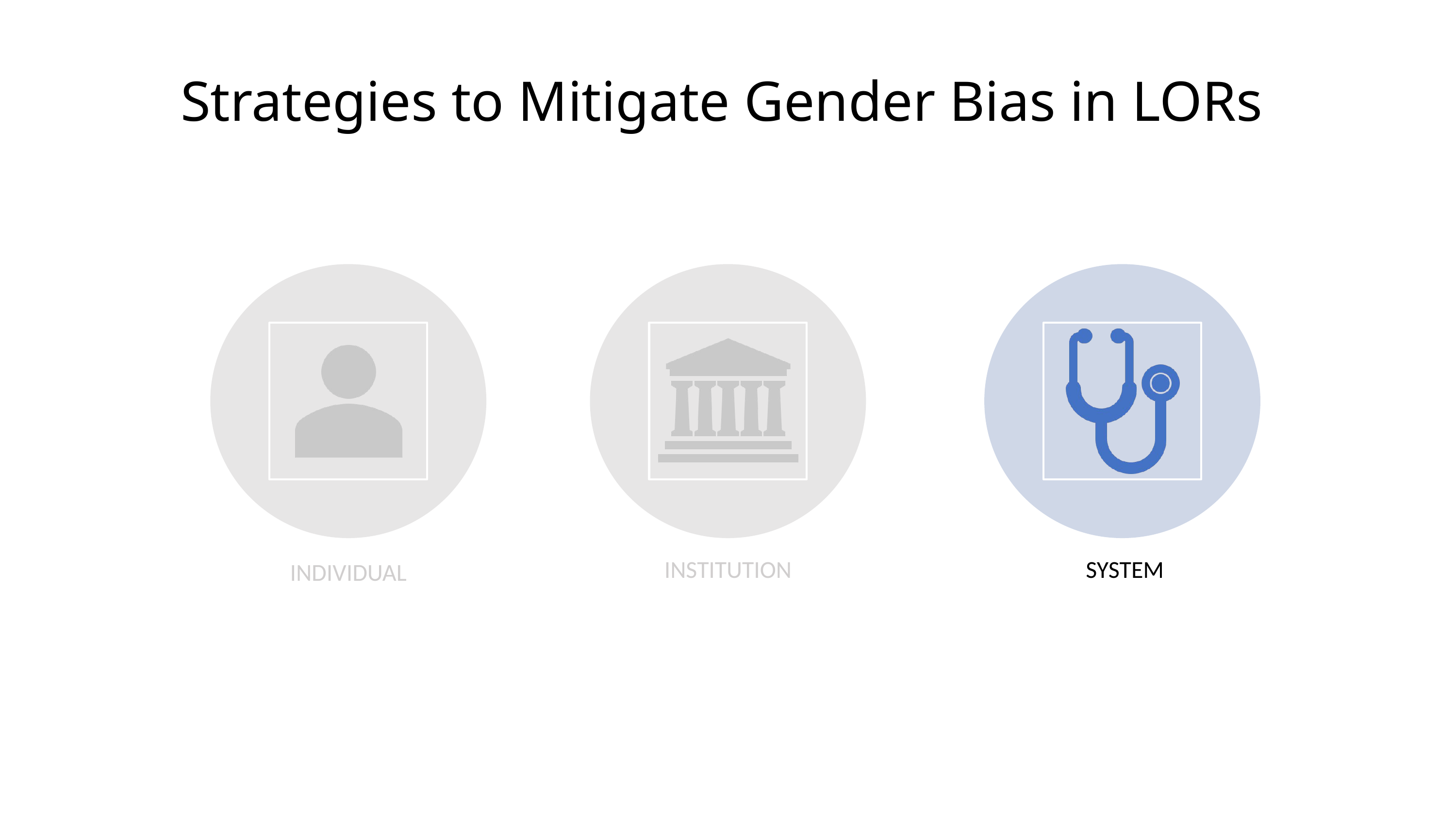

# Strategies to Mitigate Gender Bias in LORs
INSTITUTION
SYSTEM
INDIVIDUAL

## Slide 34
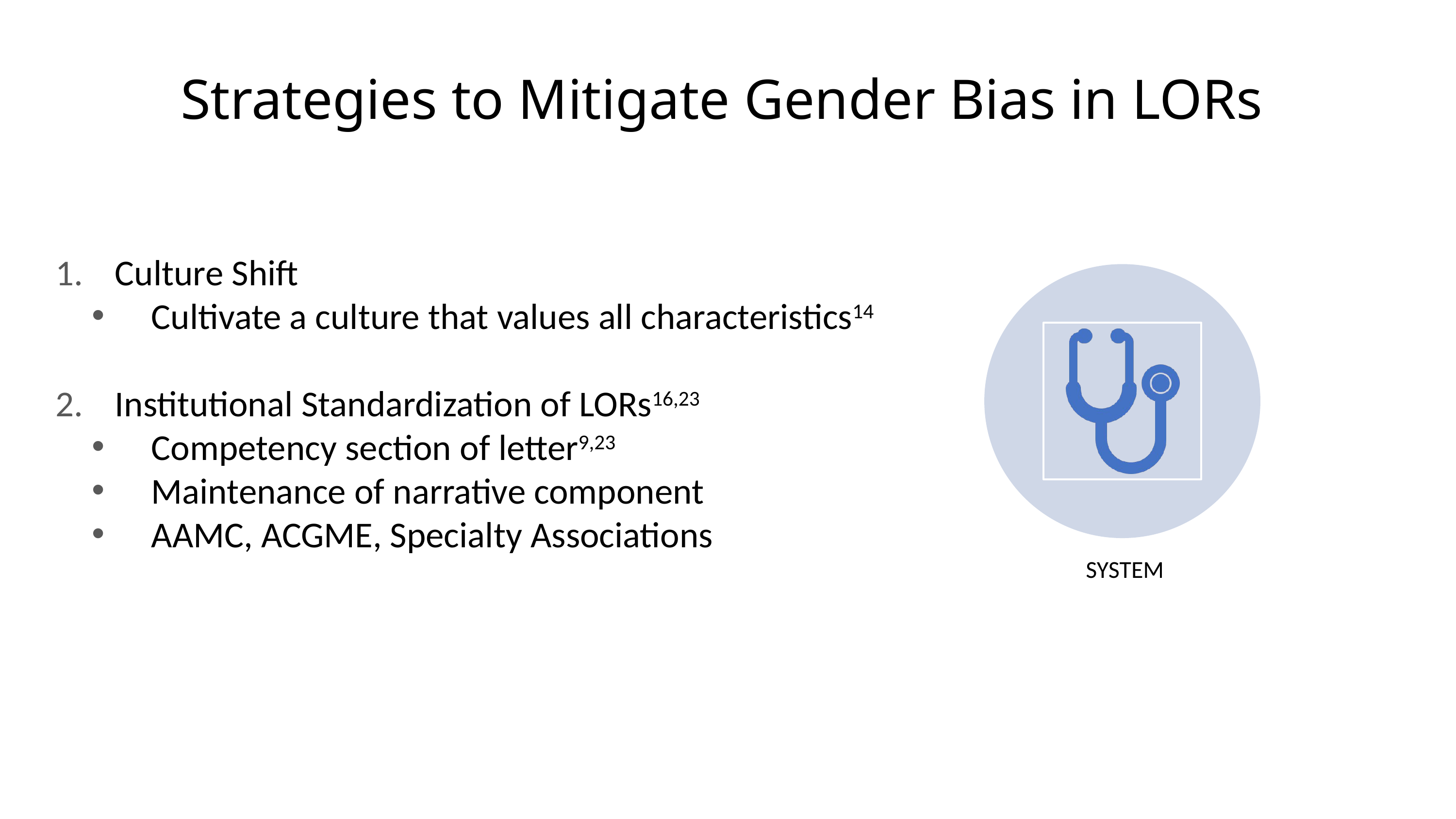

# Strategies to Mitigate Gender Bias in LORs
Culture Shift
Cultivate a culture that values all characteristics14
Institutional Standardization of LORs16,23
Competency section of letter9,23
Maintenance of narrative component
AAMC, ACGME, Specialty Associations
SYSTEM

## Slide 35
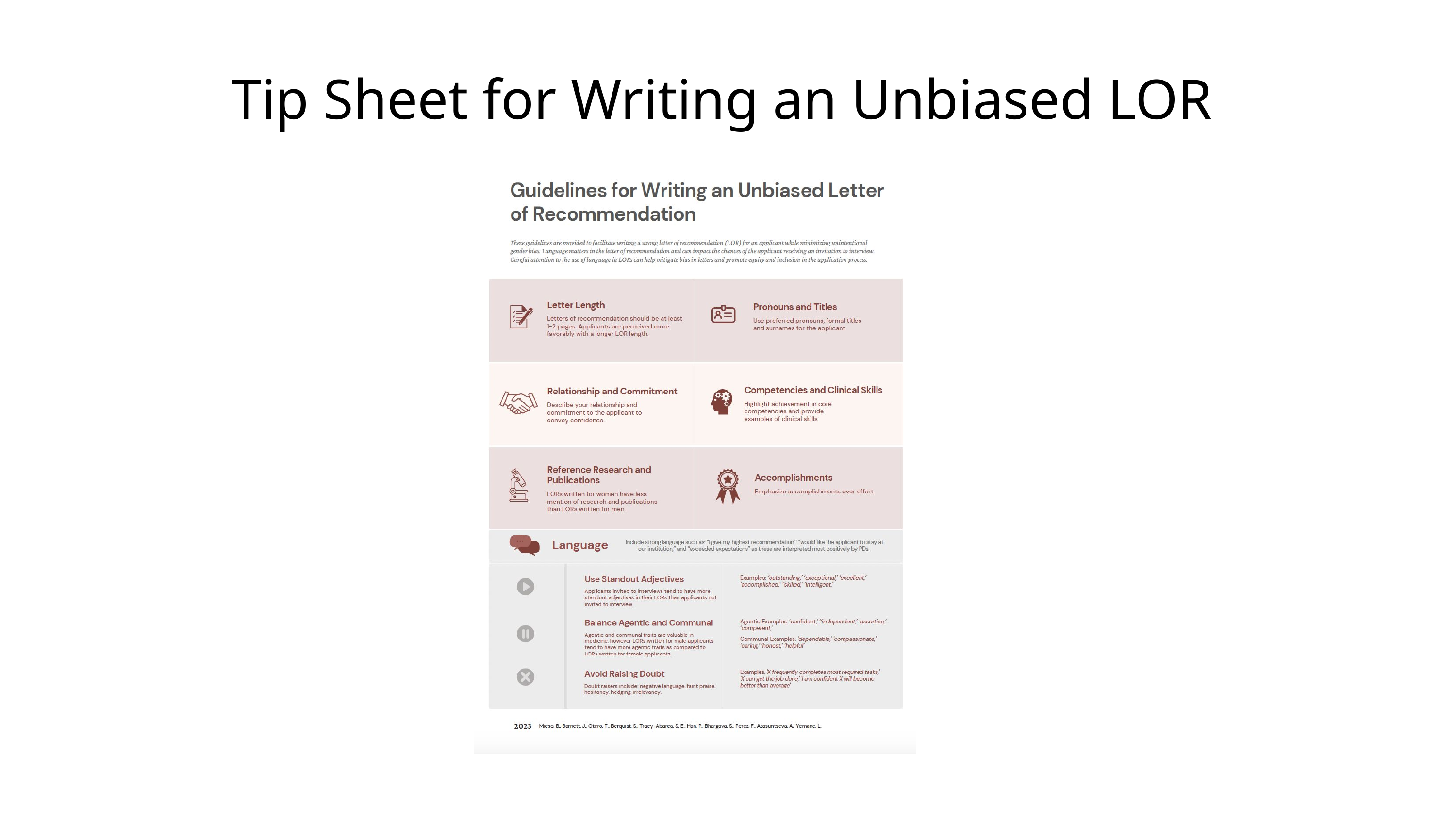

# Tip Sheet for Writing an Unbiased LOR

## Slide 36
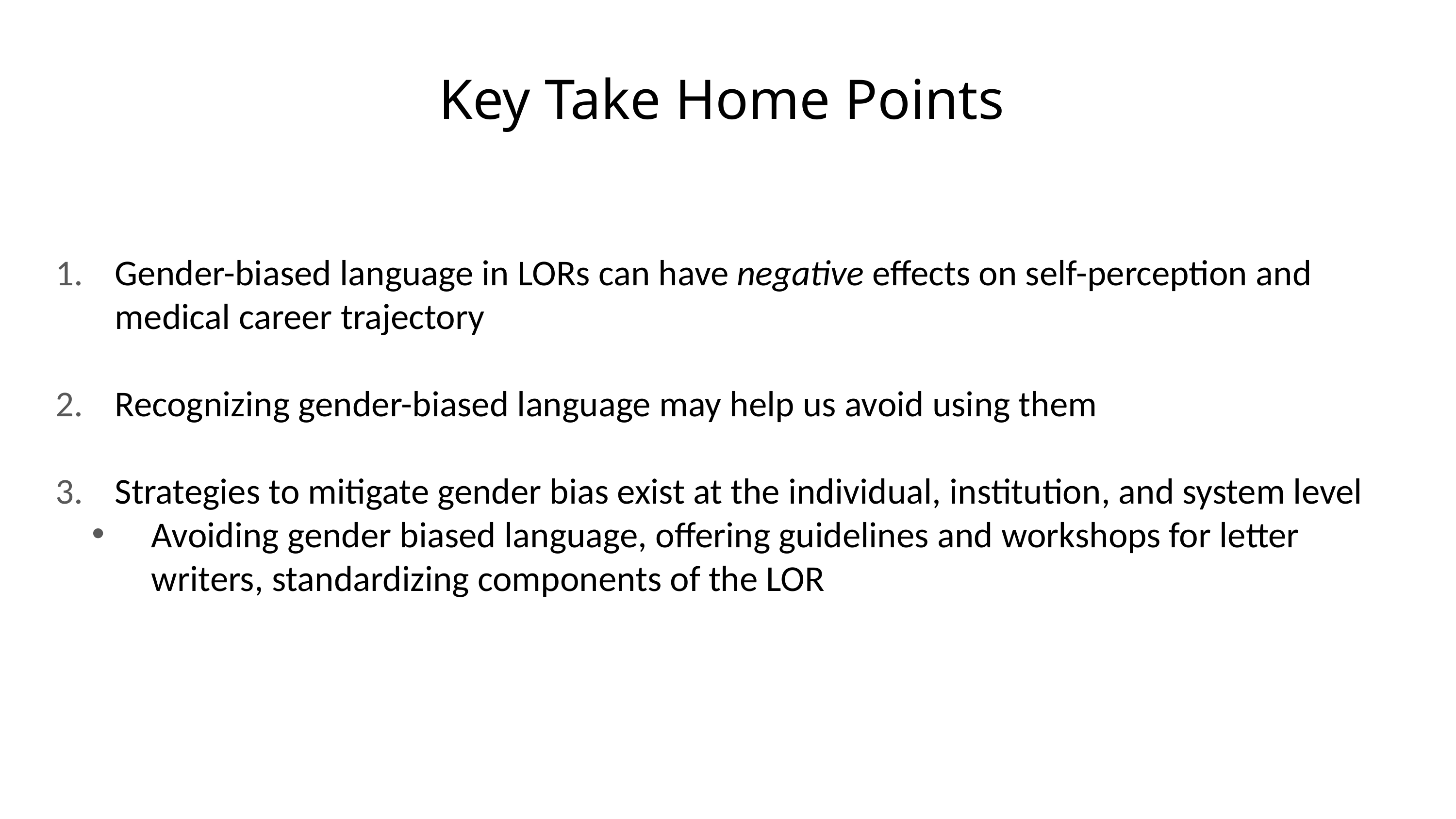

# Key Take Home Points
Gender-biased language in LORs can have negative effects on self-perception and medical career trajectory
Recognizing gender-biased language may help us avoid using them
Strategies to mitigate gender bias exist at the individual, institution, and system level
Avoiding gender biased language, offering guidelines and workshops for letter writers, standardizing components of the LOR

## Slide 37
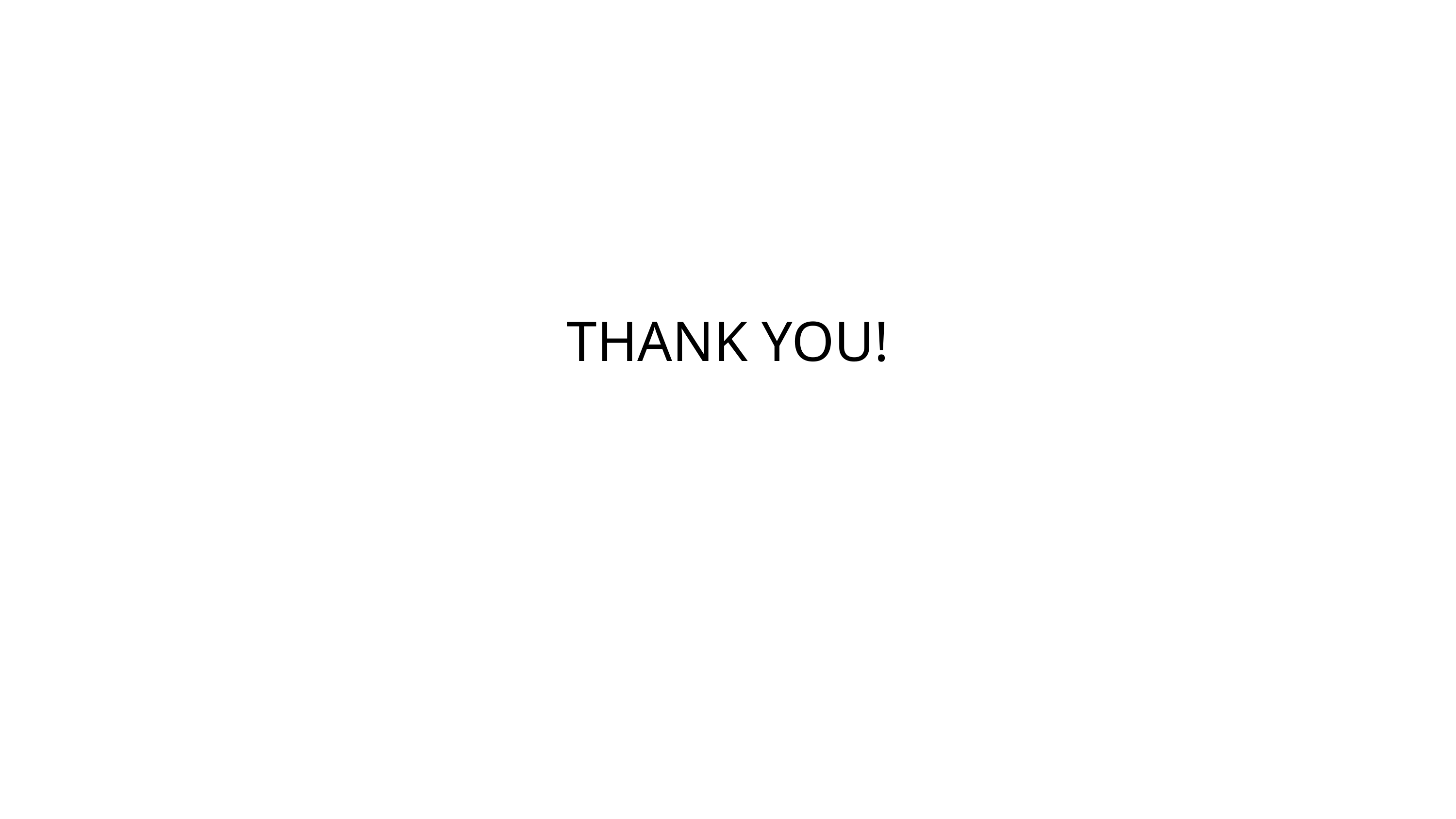

# THANK YOU!

## Slide 38
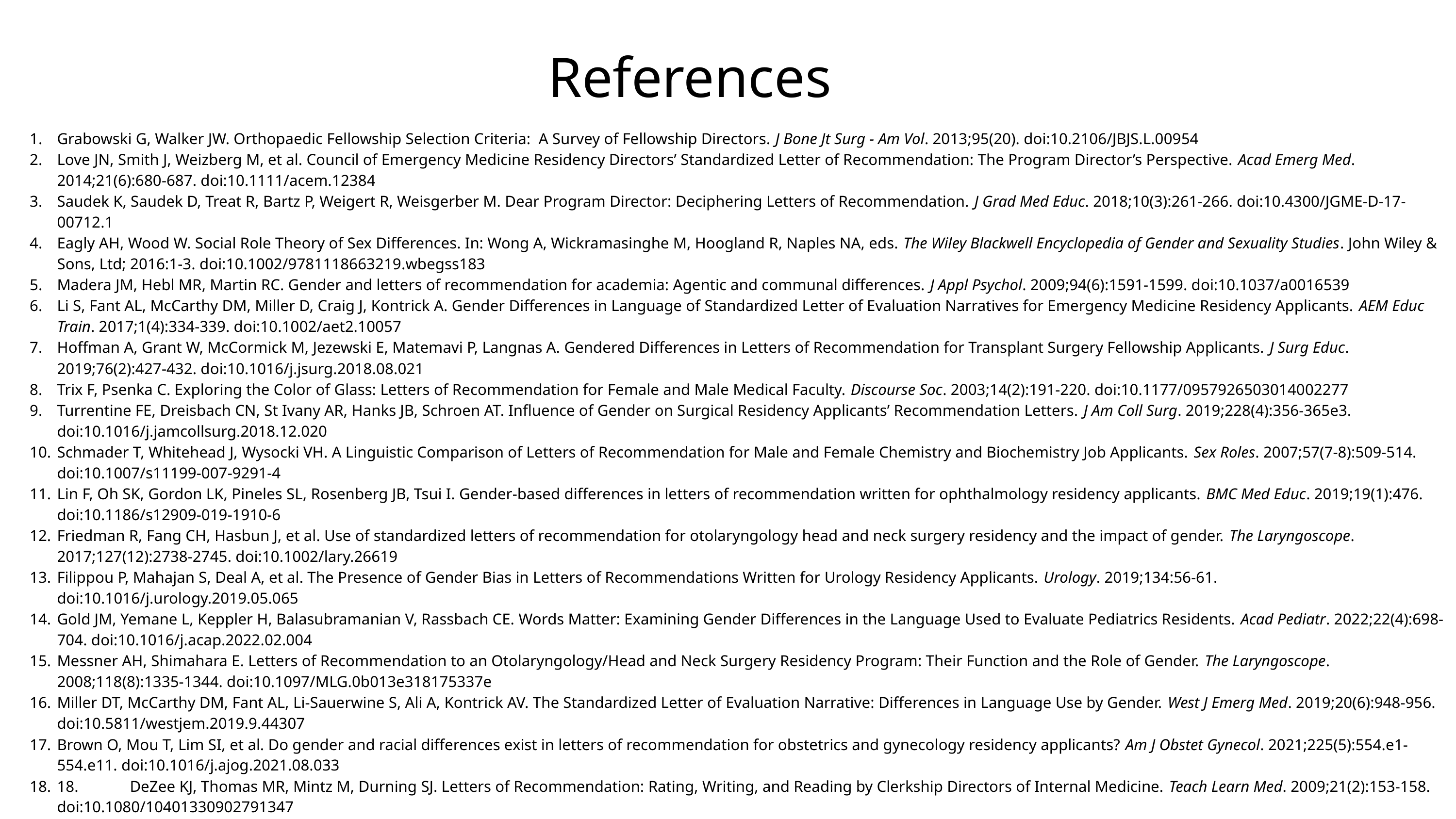

# References
Grabowski G, Walker JW. Orthopaedic Fellowship Selection Criteria: A Survey of Fellowship Directors. J Bone Jt Surg - Am Vol. 2013;95(20). doi:10.2106/JBJS.L.00954
Love JN, Smith J, Weizberg M, et al. Council of Emergency Medicine Residency Directors’ Standardized Letter of Recommendation: The Program Director’s Perspective. Acad Emerg Med. 2014;21(6):680-687. doi:10.1111/acem.12384
Saudek K, Saudek D, Treat R, Bartz P, Weigert R, Weisgerber M. Dear Program Director: Deciphering Letters of Recommendation. J Grad Med Educ. 2018;10(3):261-266. doi:10.4300/JGME-D-17-00712.1
Eagly AH, Wood W. Social Role Theory of Sex Differences. In: Wong A, Wickramasinghe M, Hoogland R, Naples NA, eds. The Wiley Blackwell Encyclopedia of Gender and Sexuality Studies. John Wiley & Sons, Ltd; 2016:1-3. doi:10.1002/9781118663219.wbegss183
Madera JM, Hebl MR, Martin RC. Gender and letters of recommendation for academia: Agentic and communal differences. J Appl Psychol. 2009;94(6):1591-1599. doi:10.1037/a0016539
Li S, Fant AL, McCarthy DM, Miller D, Craig J, Kontrick A. Gender Differences in Language of Standardized Letter of Evaluation Narratives for Emergency Medicine Residency Applicants. AEM Educ Train. 2017;1(4):334-339. doi:10.1002/aet2.10057
Hoffman A, Grant W, McCormick M, Jezewski E, Matemavi P, Langnas A. Gendered Differences in Letters of Recommendation for Transplant Surgery Fellowship Applicants. J Surg Educ. 2019;76(2):427-432. doi:10.1016/j.jsurg.2018.08.021
Trix F, Psenka C. Exploring the Color of Glass: Letters of Recommendation for Female and Male Medical Faculty. Discourse Soc. 2003;14(2):191-220. doi:10.1177/0957926503014002277
Turrentine FE, Dreisbach CN, St Ivany AR, Hanks JB, Schroen AT. Influence of Gender on Surgical Residency Applicants’ Recommendation Letters. J Am Coll Surg. 2019;228(4):356-365e3. doi:10.1016/j.jamcollsurg.2018.12.020
Schmader T, Whitehead J, Wysocki VH. A Linguistic Comparison of Letters of Recommendation for Male and Female Chemistry and Biochemistry Job Applicants. Sex Roles. 2007;57(7-8):509-514. doi:10.1007/s11199-007-9291-4
Lin F, Oh SK, Gordon LK, Pineles SL, Rosenberg JB, Tsui I. Gender-based differences in letters of recommendation written for ophthalmology residency applicants. BMC Med Educ. 2019;19(1):476. doi:10.1186/s12909-019-1910-6
Friedman R, Fang CH, Hasbun J, et al. Use of standardized letters of recommendation for otolaryngology head and neck surgery residency and the impact of gender. The Laryngoscope. 2017;127(12):2738-2745. doi:10.1002/lary.26619
Filippou P, Mahajan S, Deal A, et al. The Presence of Gender Bias in Letters of Recommendations Written for Urology Residency Applicants. Urology. 2019;134:56-61. doi:10.1016/j.urology.2019.05.065
Gold JM, Yemane L, Keppler H, Balasubramanian V, Rassbach CE. Words Matter: Examining Gender Differences in the Language Used to Evaluate Pediatrics Residents. Acad Pediatr. 2022;22(4):698-704. doi:10.1016/j.acap.2022.02.004
Messner AH, Shimahara E. Letters of Recommendation to an Otolaryngology/Head and Neck Surgery Residency Program: Their Function and the Role of Gender. The Laryngoscope. 2008;118(8):1335-1344. doi:10.1097/MLG.0b013e318175337e
Miller DT, McCarthy DM, Fant AL, Li-Sauerwine S, Ali A, Kontrick AV. The Standardized Letter of Evaluation Narrative: Differences in Language Use by Gender. West J Emerg Med. 2019;20(6):948-956. doi:10.5811/westjem.2019.9.44307
Brown O, Mou T, Lim SI, et al. Do gender and racial differences exist in letters of recommendation for obstetrics and gynecology residency applicants? Am J Obstet Gynecol. 2021;225(5):554.e1-554.e11. doi:10.1016/j.ajog.2021.08.033
18.	DeZee KJ, Thomas MR, Mintz M, Durning SJ. Letters of Recommendation: Rating, Writing, and Reading by Clerkship Directors of Internal Medicine. Teach Learn Med. 2009;21(2):153-158. doi:10.1080/10401330902791347
Alweis R, Collichio F, Milne CK, et al. Guidelines for a Standardized Fellowship Letter of Recommendation. Am J Med. 2017;130(5):606-611. doi:10.1016/j.amjmed.2017.01.017
Tavarez MM, Baghdassarian A, Bailey J, et al. A Call to Action for Standardizing Letters of Recommendation. J Grad Med Educ. 2022;14(6):642-646. doi:10.4300/JGME-D-22-00131.1
Chen BY, Hughes MT, Kern DE, Thomas PA, eds. Curriculum Development for Medical Education: A Six-Step Approach. Third edition [3rd ed.]. Johns Hopkins University Press; 2015.
Forth, T. Gender Bias Calculator. Accessed July 26, 2023. https://tomforth.co.uk/genderbias/
Zhang N, Blissett S, Anderson D, O’Sullivan P, Qasim A. Race and Gender Bias in Internal Medicine Program Director Letters of Recommendation. J Grad Med Educ. 2021;13(3):335-344. doi:10.4300/JGME-D-20-00929.1
