# Supplementary material for: Decoding the Reference Letter: Strategies to Reduce Unintentional Gender Bias in Letters of Recommendation
Source: MedEdPORTAL. 2024 Jul 5;20:11419. doi: 10.15766/mep_2374-8265.11419 (PMC11224141; doi:10.15766/mep_2374-8265.11419)
Supplement: Supplementary file 1 — Decoding the Reference Letter Presentation.pptxFacilitator Guide.docxExample Letters - Redacted Version.docxExample Letters - Unredacted Version.docxGender Bias Calculator With Example Letters.docxStanford LOR Tip Sheet.pdfWorkshop Evaluation Form.doc [file mep_2374-8265.11419-s001.zip › B. Facilitator Guide.docx]

**Decoding the Reference Letter: Strategies to Reduce Unintentional Gender Bias in Letters of Recommendation**

*Facilitator Guide*

**Facilitator Guide Contents**

- Educational Objectives
- Pre-Reading Assignments for Facilitators
- Supplies Needed
- Workshop Agenda

**Educational Objectives**

By the end of this workshop, participants will be able to:

1. Define linguistic bias and gender bias in letters of recommendation (LORs)
2. Identify adjectives in LORs that are commonly associated with gender bias
3. Apply tools learned to mitigate gender bias when writing LORs​
4. Discuss strategies to reduce gender bias when writing LORs

**Pre-Reading Assignments for Facilitators**

1. Trix F, Psenka C. Exploring the Color of Glass: Letters of Recommendation for Female and Male Medical Faculty. *Discourse Soc*. 2003;14(2):191-220. doi:10.1177/0957926503014002277
2. Madera JM, Hebl MR, Martin RC. Gender and letters of recommendation for academia: Agentic and communal differences. *J Appl Psychol*. 2009;94(6):1591-1599. doi:10.1037/a0016539
3. Zhang N, Blissett S, Anderson D, O’Sullivan P, Qasim A. Race and Gender Bias in Internal Medicine Program Director Letters of Recommendation. *J Grad Med Educ*. 2021;13(3):335-344. doi:10.4300/JGME-D-20-00929.1

**Supplies Needed:**

1. PowerPoint Slides (Appendix A)
2. Facilitator Guide (Appendix B)
3. Example Letters - Redacted Version (Appendix C)
4. Example Letters - Unredacted Version (Appendix D)
5. Gender Bias Calculator with Example Letters (Appendix E)
6. Stanford LOR Tip Sheet (Appendix F)
7. Workshop Evaluation Form (Appendix G)

*Note:* Consider providing supplementary handouts for Appendices C-E, which mirror the PowerPoint slides, to enhance overall readability if font on slides is too small for participants or presentation is given in a large room.

**Workshop Agenda**

*60 Minute Version (Virtual)*

- **Introduction:** 3 minutes
  - Introduction (1 min)
  - Review educational objectives (1 min)
  - Disclosures and disclaimer about other forms of bias (1 min)
- **Reflection Activity - LORs:** 6 minutes
  - Compare and contrast two excerpts of LORs (names and pronouns redacted) (4 min)
  - Share observations in chat box (2 min)
- **Brief Didactic on Relevance of Topic:** 6 minutes
  - Discuss the impact of gender stereotypes (3 min)
  - Explain the importance of this topic (3 min)
- **Activity and Reflection - Agentic and Communal Traits:** 10 minutes
  - Participants asked to share 3 personal characteristics in the chat (1 min)
  - Introduce the concepts of agentic and communal traits (2 min)
  - Participants asked to categorize the shared characteristics as either agentic or communal and reflect in the chat box and/or using the microphone (7 min)
- **Individual Strategies to Mitigate Gender Bias:** 25 minutes
  - Presentation: 10 minutes
    - Individual Strategies (1 min)
    - Review forms of gender bias in LORs as a way to avoid them on an individual level (9 min)
- **Large Group Activity and Reflection:** 15 minutes
  - Introduce the gender bias calculator tool (1 min)
  - Review excerpts of LORs from the initial reflection activity now with names and pronouns revealed; compare and contrast (8 min)
  - Compare and contrast the excerpts of LORs using the gender bias calculator (1 min)
  - Reflect on the utility of the gender bias calculator using the chat box and/or using the microphone (5 min)
- **Institutional and Systemic Strategies to Mitigate Gender Bias:** 5 minutes
  - Disseminate Stanford LOR Tip Sheet
- **Conclusions:** 5 minutes
  - Provide key take home points, opportunity for Q&A, disseminate and complete evaluation form

**Workshop Agenda**

*60 Minute Version (In-Person)*

- **Introduction:** 3 minutes
  - Introduction (1 min)
  - Review educational objectives (1 min)
  - Disclosures and disclaimer about other forms of bias (1 min)
- **Reflection Activity - LORs:** 6 minutes
  - Compare and contrast two excerpts of LORs (names and pronouns redacted) in small groups (4 min)
  - Share observations in large group (2 min)
- **Brief Didactic on Relevance of Topic:** 6 minutes
  - Discuss the impact of gender stereotypes (3 min)
  - Explain the importance of this topic (3 min)
- **Activity and Reflection - Agentic and Communal Traits:** 10 minutes
  - Participants asked to share 3 personal characteristics on sticky notes provided (1 min)
  - Introduce the concepts of agentic and communal traits (2 min)
  - Participants asked to categorize the shared characteristics as either agentic or communal and post the sticky notes into the column that best fits the characteristic (5 min)
  - Participants asked to share reflections in large group (2 min)
- **Individual Strategies to Mitigate Gender Bias:** 25 minutes
  - Presentation: 10 minutes
    - Individual Strategies (1 min)
    - Review forms of gender bias in LORs as a way to avoid them on an individual level (9 min)
- **Large Group Activity and Reflection:** 15 minutes
  - Introduce the gender bias calculator tool (1 min)
  - Review excerpts of LORs from the initial reflection activity now with names and pronouns revealed; compare and contrast (8 min)
  - Compare and contrast the excerpts of LORs using the gender bias calculator (1 min)
  - Reflect on the utility of the gender bias calculator in large group discussion (5 min)
- **Institutional and Systemic Strategies to Mitigate Gender Bias:** 5 minutes
  - Disseminate Stanford LOR Tip Sheet
- **Conclusions:** 5 minutes
  - Provide key take home points, opportunity for Q&A, disseminate and complete evaluation form
