# Supplementary material for: Decoding the Reference Letter: Strategies to Reduce Unintentional Gender Bias in Letters of Recommendation
Source: MedEdPORTAL. 2024 Jul 5;20:11419. doi: 10.15766/mep_2374-8265.11419 (PMC11224141; doi:10.15766/mep_2374-8265.11419)
Supplement: Supplementary file 1 — Decoding the Reference Letter Presentation.pptxFacilitator Guide.docxExample Letters - Redacted Version.docxExample Letters - Unredacted Version.docxGender Bias Calculator With Example Letters.docxStanford LOR Tip Sheet.pdfWorkshop Evaluation Form.doc [file mep_2374-8265.11419-s001.zip › C. Example Letters - Redacted Version.docx]

**Example #1:** It is my pleasure to recommend Mr. Nicholas Holmes to you as an exceptional candidate for your residency program. I worked closely with Mr. Holmes during h is acting internship. H e demonstrates excellent clinical skills and medical knowledge. H is clinical judgment is outstanding. H e has a unique ability to effectively communicates with patients, families, and the care team. H e has published several works and has received numerous awards for his research in cardiology…*[ +45 lines].* I recommend him without reservation to your program.

**Example #2:** I am writing this letter of recommendation on behalf of Alyssa Sims. Ms. Sims is hardworking, caring, dedicated, and compassionate toward her patients. She has an appropriate fund of knowledge. She is a dependable team member and diligent in completing patient tasks. *[+20 lines].*I recommend her to your program.
