# Supplementary material for: Decoding the Reference Letter: Strategies to Reduce Unintentional Gender Bias in Letters of Recommendation
Source: MedEdPORTAL. 2024 Jul 5;20:11419. doi: 10.15766/mep_2374-8265.11419 (PMC11224141; doi:10.15766/mep_2374-8265.11419)
Supplement: Supplementary file 1 — Decoding the Reference Letter Presentation.pptxFacilitator Guide.docxExample Letters - Redacted Version.docxExample Letters - Unredacted Version.docxGender Bias Calculator With Example Letters.docxStanford LOR Tip Sheet.pdfWorkshop Evaluation Form.doc [file mep_2374-8265.11419-s001.zip › D. Example Letters - Unredacted Version.docx]

**Example #1:** It is my pleasure to recommend ***Mr. Nicholas Holmes*** to you as an ***exceptional*** candidate for your residency program. I ***worked closely*** with Mr. Holmes during his acting internship. He demonstrates ***excellent*** clinical skills and medical knowledge. His clinical judgment is ***outstanding***. He has a unique ability to effectively communicates with patients, families, and the care team. He has ***published several works*** and has received numerous ***awards*** for his ***research*** in cardiology**…*[ +45 lines].*** I ***recommend him without reservation*** to your program.

**Example #2:** I am writing this letter of recommendation on behalf of ***Alyssa Sims***. Ms. Sims is ***hardworking, caring, dedicated, and compassionate*** toward her patients. She has an ***appropriate fund of knowledge***. She is a ***dependable*** team member and ***diligent in completing patient tasks*…*[+20 lines****].****I recommend her*** to your program.
