# Supplementary material for: Decoding the Reference Letter: Strategies to Reduce Unintentional Gender Bias in Letters of Recommendation
Source: MedEdPORTAL. 2024 Jul 5;20:11419. doi: 10.15766/mep_2374-8265.11419 (PMC11224141; doi:10.15766/mep_2374-8265.11419)
Supplement: Supplementary file 1 — Decoding the Reference Letter Presentation.pptxFacilitator Guide.docxExample Letters - Redacted Version.docxExample Letters - Unredacted Version.docxGender Bias Calculator With Example Letters.docxStanford LOR Tip Sheet.pdfWorkshop Evaluation Form.doc [file mep_2374-8265.11419-s001.zip › F. Stanford LOR Tip Sheet.pdf]

Guidelines for Writing an Unbiased Letter of Recommendation

These guidelines are provided to facilitate writing a strong letter of recommendation (LOR) for an applicant while minimizing unintentional gender bias. Language matters in the letter of recommendation and can impact the chances of the applicant receiving an invitation to interview. Careful attention to the use of language in LORs can help mitigate bias in letters and promote equity and inclusion in the application process.

Letter Length

Letters of recommendation should be at least 1-2 pages. Applicants are perceived more favorably with a longer LOR length.

Pronouns and Titles

Use preferred pronouns, formal titles and surnames for the applicant.

Relationship and Commitment

Describe your relationship and commitment to the applicant to convey confidence.

Competencies and Clinical Skills

Highlight achievement in core competencies and provide examples of clinical skills.

Reference Research and Publications

LORs written for women have less mention of research and publications than LORs written for men.

Accomplishments

Emphasize accomplishments over effort.

Language

Include strong language such as: "I give my highest recommendation," "would like the applicant to stay at our institution," and "exceeded expectations" as these are interpreted most positively by PDs.

Use Standout Adjectives

Applicants invited to interviews tend to have more standout adjectives in their LORs than applicants not invited to interview.

Examples: *'outstanding,' 'exceptional,' 'excellent,' 'accomplished,' "skilled,' 'intelligent,'*

Balance Agentic and Communal

Agentic and communal traits are valuable in medicine, however LORs written for male applicants tend to have more agentic traits as compared to LORs written for female applicants.

Agentic Examples: 'confident,' *''independent,' 'assertive,' 'competent,'*

Communal Examples: *'dependable,' 'compassionate,' 'caring,' 'honest,' 'helpful'*

Avoid Raising Doubt

Doubt raisers include: negative language, faint praise, hesitancy, hedging, irrelevancy.

Examples: *'X frequently completes most required tasks,' 'X can get the job done,' 'I am confident X will become better than average'*

2023 Mieso, B., Barnett, J., Otero, T., Berquist, S., Tracy-Abarca, S. E., Han, P., Bhargava, S., Perez, F., Atasuntseva, A., Yemane, L.
