# Supplementary figures and images for: Decoding the Reference Letter: Strategies to Reduce Unintentional Gender Bias in Letters of Recommendation
Source: MedEdPORTAL. 2024 Jul 5;20:11419. doi: 10.15766/mep_2374-8265.11419 (PMC11224141; doi:10.15766/mep_2374-8265.11419)

Example #1


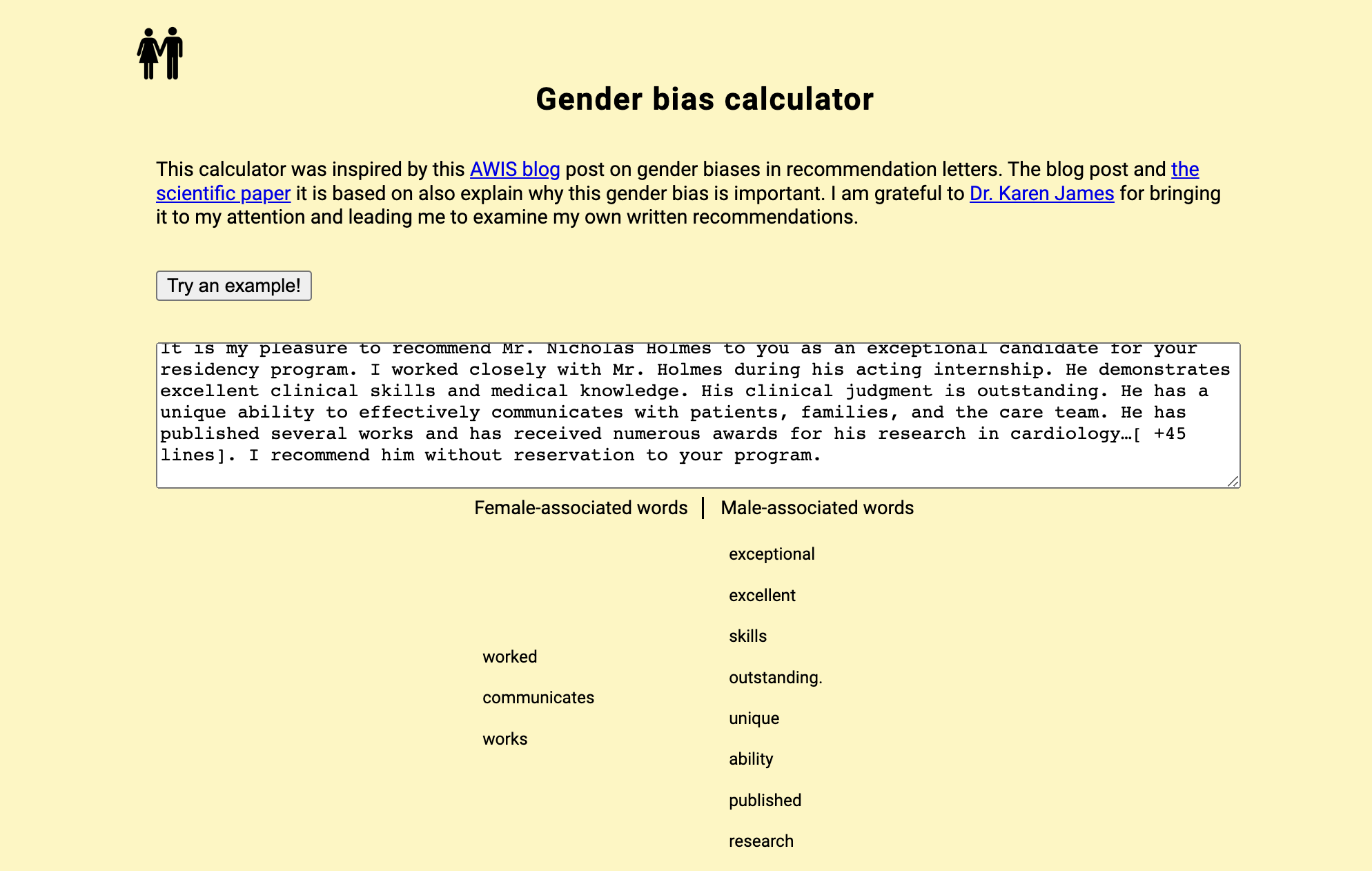


Example #2


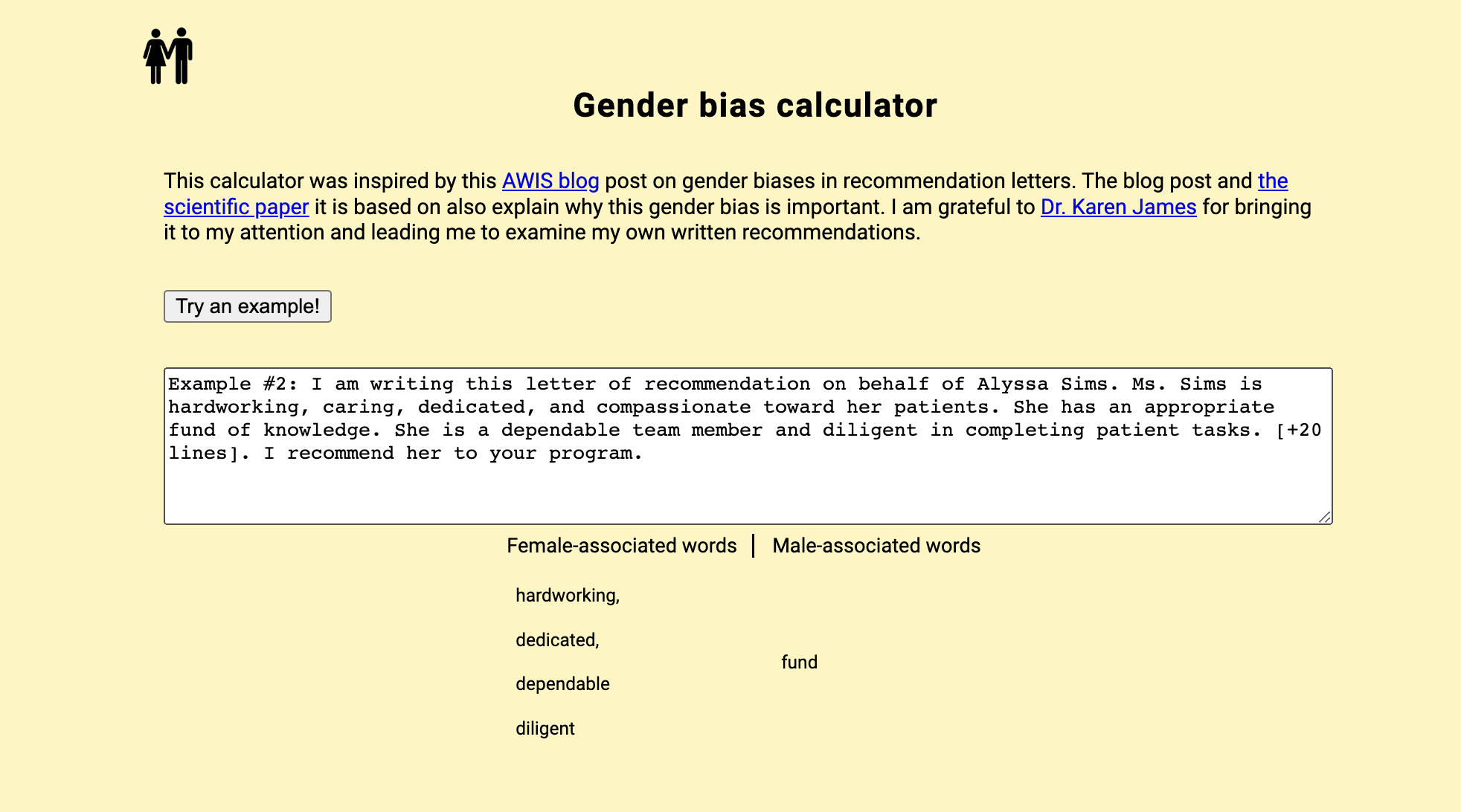

Supplement: Supplementary file 1 — Decoding the Reference Letter Presentation.pptxFacilitator Guide.docxExample Letters - Redacted Version.docxExample Letters - Unredacted Version.docxGender Bias Calculator With Example Letters.docxStanford LOR Tip Sheet.pdfWorkshop Evaluation Form.doc [file mep_2374-8265.11419-s001.zip › E. Gender Bias Calculator With Example Letters.docx]
