# Supplementary material for: Decoding the Reference Letter: Strategies to Reduce Unintentional Gender Bias in Letters of Recommendation
Source: MedEdPORTAL. 2024 Jul 5;20:11419. doi: 10.15766/mep_2374-8265.11419 (PMC11224141; doi:10.15766/mep_2374-8265.11419)
Supplement: Supplementary file 1 — Decoding the Reference Letter Presentation.pptxFacilitator Guide.docxExample Letters - Redacted Version.docxExample Letters - Unredacted Version.docxGender Bias Calculator With Example Letters.docxStanford LOR Tip Sheet.pdfWorkshop Evaluation Form.doc [file mep_2374-8265.11419-s001.zip › G. Workshop Evaluation Form.doc]

**LEAD Workshop:** Decoding the Reference Letter: Strategies to Reduce Unintentional Gender Bias in Letters of Recommendation

**[Date]**

**EVALUATION FORM**

Strongly disagree	Neutral	Strongly agree

Workshop met objective #1:	1	2	3	4	5

Define linguistic bias and gender bias in letters of recommendation (LORs)

Workshop met objective #2:	1	2	3	4	5

Identify adjectives in LORs that are commonly associated with gender bias

Workshop met objective #3:	1	2	3	4	5

Apply tools learned to mitigate gender bias when writing LORs

Workshop met objective #4:	1	2	3	4	5

Discuss strategies to reduce gender bias when writing LORs

Workshop was a valuable use of my time	1	2	3	4	5

Handouts include useful resources	1	2	3	4	5

I will apply information learned today	1	2	3	4	5

What are two things you will do differently as a result of this workshop?

What do you see as potential barriers to applying what you have learned?

What did you like best about the workshop?

What can we improve about the workshop?
